# Improved prognostic classification of breast cancer defined by antagonistic activation patterns of immune response pathway modules

**DOI:** 10.1186/1471-2407-10-604

**Published:** 2010-11-04

**Authors:** Andrew E Teschendorff, Sergio Gomez, Alex Arenas, Dorraya El-Ashry, Marcus Schmidt, Mathias Gehrmann, Carlos Caldas

**Affiliations:** 1 Breast Cancer Functional Genomics Laboratory, Cancer Research UK Cambridge Research Institute and Department of Oncology University of Cambridge, Li Ka-Shing Centre, Robinson Way, Cambridge CB2 0RE, UK; 2 Departament d'Enginyeria Informatica i Matematiques, Universitat Rovira i Virgili, 43007 Tarragona, Spain; 3 Institute for Biocomputation and Physics of Complex Systems (BIFI), University of Zaragoza, Zaragoza 50009, Spain; 4 Lawrence Berkeley National Laboratory, Berkeley, CA 94720, USA; 5 Sylvester Comprehensive Cancer Center and Braman Family Breast Cancer Institute, University of Miami Miller School of Medicine, Miami FL 33136, USA; 6 Department of Obstetrics and Gynecology, Medical School, Johannes Gutenberg University, Mainz 55131, Germany; 7 Siemens Medical Solutions Diagnostics GmbH, Cologne 50829, Germany; 8 Medical Genomics Group, Paul O'Gorman Building, UCL Cancer Institute, University College London, 72 Huntley Street, London WC1E 6BT, UK

## Abstract

**Background:**

Elucidating the activation pattern of molecular pathways across a given tumour type is a key challenge necessary for understanding the heterogeneity in clinical response and for developing novel more effective therapies. Gene expression signatures of molecular pathway activation derived from perturbation experiments in model systems as well as structural models of molecular interactions ("model signatures") constitute an important resource for estimating corresponding activation levels in tumours. However, relatively few strategies for estimating pathway activity from such model signatures exist and only few studies have used activation patterns of pathways to refine molecular classifications of cancer.

**Methods:**

Here we propose a novel network-based method for estimating pathway activation in tumours from model signatures. We find that although the pathway networks inferred from cancer expression data are highly consistent with the prior information contained in the model signatures, that they also exhibit a highly modular structure and that estimation of pathway activity is dependent on this modular structure. We apply our methodology to a panel of 438 estrogen receptor negative (ER-) and 785 estrogen receptor positive (ER+) breast cancers to infer activation patterns of important cancer related molecular pathways.

**Results:**

We show that in ER negative basal and HER2+ breast cancer, gene expression modules reflecting T-cell helper-1 (Th1) and T-cell helper-2 (Th2) mediated immune responses play antagonistic roles as major risk factors for distant metastasis. Using Boolean interaction Cox-regression models to identify non-linear pathway combinations associated with clinical outcome, we show that simultaneous high activation of Th1 and low activation of a TGF-beta pathway module defines a subtype of particularly good prognosis and that this classification provides a better prognostic model than those based on the individual pathways. In ER+ breast cancer, we find that simultaneous high MYC and RAS activity confers significantly worse prognosis than either high MYC or high RAS activity alone. We further validate these novel prognostic classifications in independent sets of 173 ER- and 567 ER+ breast cancers.

**Conclusion:**

We have proposed a novel method for pathway activity estimation in tumours and have shown that pathway modules antagonize or synergize to delineate novel prognostic subtypes. Specifically, our results suggest that simultaneous modulation of T-helper differentiation and TGF-beta pathways may improve clinical outcome of hormone insensitive breast cancers over treatments that target only one of these pathways.

## Background

A key challenge to improve our understanding of the heterogeneity in clinical outcome and response to therapy is to map out the activation levels of cancer-relevant pathways across clinical tumour specimens. To address this goal, some studies have begun to characterise oncogenic and cancer-signalling pathways in terms of "gene expression signatures", typically derived from perturbation experiments that were performed *in-vitro *or in model-systems, and in which specific signalling was either enhanced or inhibited [1-4]. Most of the genes that make up these perturbation signatures do not coincide with those involved in the primary cascades following the perturbation (as given for example by the local protein-interaction network surrounding the perturbation) [5]. Instead, most of the genes in these signatures reflect downstream transcriptional consequences of the perturbation, which may nevertheless provide better measures of upstream pathway activity [5]. Other studies have focused on using literature curated databases of molecular pathway interactions, thus taking the alternative view that consistency and trends in mRNA expression levels of interacting proteins may be used to infer pathway activity [6-8]. In this work we refer to both the perturbation signatures and molecular interaction models as "model signatures". These same studies and others have also begun to explore the clinical relevance of such model signatures by inferring pathway activity across human tumours and correlating the inferred patterns with clinical variables [1,6,7,9-14]. As the studies in [5,14] suggest, using molecular pathways may offer the potential to delineate novel clinically relevant subtypes within heterogeneous cancers.

Breast cancer patients with same histopathological features demonstrate wide differences in clinical outcome. For example, despite the aggressive high grade nature of ER- disease, not all ER- patients have a poor clinical outcome and a molecular subgroup of good prognosis was recently identified in [15,16]. This subgroup was characterised by overexpression of an immune-response gene module and others have since reported similar findings [17-23]. These results strongly implicate tumor stromal cells, including T-cells and macrophages, as molecular determinants of clinical outcome in breast cancer [21]. However, results have also been mixed with reports of inverse associations of immune response genes with good prognosis, partly dependent on ER status [18,24], and which have obscured the role of immune cells in prognosis. More recently, it has been shown that T-cell helper-2 (Th2) mediated immune response pathways may promote tumor metastasis in mammary carcinomas, in contrast to T-cell helper-1 (Th1) immune response pathways which are thought to be tumor inhibitory [25,26]. In spite of this growing interest in understanding the role of immune response pathways in breast cancer, to date no study has investigated if these pro and antimetastatic behaviours are reflected in bulk tumour mRNA expression profiles and how these relate to clinical outcome.

In view of this, we decided to take a bioinformatic approach to dissect bulk tumour gene expression profiles in terms of model pathway signatures, in order to shed further light on the prognostic role of immune response and other important molecular pathways in breast cancer. While statistical methods for inferring pathway activation levels from corresponding model signatures have been proposed [2,5,6,27-29], it has recently become clear that model signatures exhibit a highly complex modular structure that needs to be factored in when estimating pathway activity [5]. For example, given the genes that are coordinately up and downregulated upon oncogene activation in a cell-line, not all of these may demonstrate the same coherent up and down regulatory pattern in a tumour sample that has this oncogene activated. This may be because of other perturbations (mutations) present in that tumour, tumour cell heterogeneity, differences caused by the tumour microenvironment, or because of inherent cross-talk between molecular pathways. Motivated by these difficulties, we propose a modular approach to pathway estimation using ideas and methods from network topology [30-32]. Unlike the clustering and factor analysis approaches of [2,5,27,28], we allow the information content of a model signature to be evaluated against its expression pattern across a large panel of tumour samples, thus allowing the consistency and relevance of the model in the different cellular context to be established before estimating module activity. The evaluation of pathway consistency and activity scores was also an approach used in [8]. Recent studies have also shown the added value of using network based approaches [6,33,34] and large expression compendia [33-37] to derive gene modules associated with specific cancer phenotypes. The work presented here differs from most of these studies in that (i) our network approach is totally unsupervised and (ii) that we tackle the specific problem of pathway module activity estimation without reference to a particular phenotype.

The main contributions of this manuscript are two-fold. First, we propose a novel graph-theory framework for obtaining pathway module activity estimates and demonstrate the consistency of the method. Second, we apply it to estimate activation levels of modules within a number of important molecular pathways (HRAS, E2F3, MYC, ERBB2, EGFR, AKT, IL12, IL2, IL4, IL13, IFNG, TGFB) (Methods) [1,3,11,25,38,39] in ER+ and ER- breast cancer and show that specific pathway modules synergize to provide better prognostic stratifications of tumour samples. Specifically, we demonstrate that ER- tumours characterised by simultaneous high activation of a Th-1 differentiation module and low activation of a TGFB pathway module have better clinical outcome than tumours stratified by each pathway alone. Thus, estimating pathway module activity levels and considering models of combined pathway activation to delineate novel prognostic subtypes may hold promise as a general technique for proposing novel and more effective combinational therapies.

## Methods

### Data sets and molecular pathways

Central to our strategy is the availability of a large data set in order to ascertain the most robust gene-gene correlations. We constructed a large expression set by merging seven of the largest breast cancer data sets together [2,24,40-44]. These data sets were chosen because of their size, quality and available clinical outcome information. The normalised data provided by the authors was used and only probes that mapped to NCBI Entrez ID identifiers selected. Probes mapping to the same Entrez ID identifiers were averaged. We found 6265 genes in common between the seven studies. Samples in each study were then divided up into estrogen receptor negative and positive (ER-, ER+) tumours based on available immunohistochemical information. This division was necessary for the subsequent merging procedure to work, because cohorts differed substantially in terms of the relative proportions of ER- and ER+ tumors and because ER- and ER+ tumors show widely different gene expression profiles [40,41]. Next, for each set of ER+ and ER- tumours within a study, we renormalised the gene expression profile by a mean centering and scaling the standard deviation to 1, yielding the z-score expression profile. For each common gene, z-scores were then merged across all ER- cohorts, and similarly for all ER+ cohorts. This merging procedure was already validated and shown to be a very fruitful approach [15,45]. For the seven cohorts this yielded two large mRNA expression data sets of ER+ (785 samples) and ER- (438 samples) tumours over a common set of 6265 genes. We validated the merging by performing a Singular Value Decomposition (SVD) and demonstrating that none of the top 20 singular values correlated significantly with the cohort of origin, while correlating significantly with known intrinsic subtypes.

To assign intrinsic (SSP) subtypes within each study we used spearman correlations between the intrinsic centroids and the sample expression profiles followed by a nearest centroid criterion [46]. This was done by mapping the genes in the centroids to the corresponding averaged gene profiles on each indidividual platform (i.e we considered the overlap with all genes in each study and not just those overlapping the 6265 common genes).

We selected a number of molecular pathways with important roles in breast cancer: Ha-Ras1 proto-oncoprotein (HRAS), E2F transcription factor 3 (E2F3) and c-myc proto-oncogene protein (MYC) [1], epidermal growth factor receptor (EGFR) and c-erb B2/neu protein (ERBB2) [3], AKT1 kinase (AKT) [11], interferon-gamma (IFNG) [39], transforming growth factor beta-1 (TGFB) [38], interleukin-2,4,12,13 (IL-2,4,12,13) (*http://stke.sciencemag.org/, http://www.biocarta.com/*, [25]). For the HRAS, E2F3, MYC, EGFR and ERBB2 pathways we used as model signatures the perturbation signatures of up and down regulation reported in the respective publications [1,3]. These signatures were derived from human mammary epithelial cells and are reflective of the perturbations in the respective oncogenes in independent data [1,3]. For the pathways representing (AKT,IFNG,TGFB) we used as model signatures the genes reported to be upregulated upon activation of the pathway [11,38,39] and which are part of the highly curated Molecular Signatures Database (*http://www.broadinstitute.org/gsea/msigdb/*). For the cytokine pathways we used the highly curated pathway databases (*http://stke.sciencemag.org/, http://www.biocarta.com/*) to identify genes which are normally upregulated in response to these cytokines. The genes and their directionality of regulation in each pathway are provided in Additional file 1.

### Rationale for a modular approach

Proposed methods for estimating pathway activity differ mainly in terms of the amount of information contained in the model signature that is subsequently used for pathway activity estimation. In the simplest approach, the model signature is treated as a gene-list and proceeds by clustering the genes across clinical tumour samples to then infer activity scores over the separate clusters [2]. The advantage of this approach is that it is very plastic in that it recognises that a model signature will break up into clusters or modules once the pattern of expression of the constituent genes is investigated in a different biological context. On the other hand, a potential disadvantage is that it doesn't use all the information content in the model signature and thus does not evaluate the consistency of the model signature in the different context prior to pathway estimation. This "clustering approach" contrasts with the Bayesian regression approach, where activity levels are estimated by computing correlation-like scores between PCA components inferred from a training set and the expression profile of any given sample [27,28]. While a clear advantage of the regression approach is that it makes use of all the information content of the model signature, it is much less plastic as it implicitly assumes that all of the genes and weights in the model signature are relevant for estimating the activation of the corresponding pathway in the different cellular context. Even if model signatures are inferred by carefully avoiding overfitting in the training process, this would only avoid overfitting if the "test" set samples were of the same characteristics as the training samples, a condition which is often not satisfied. To address this problem, a modular approach like the ones used in [2,5] seems necessary, as such methods recognise that not all genes in the model signature are relevant for pathway estimation.

In the case of prognostic signatures in ER+ breast cancer, as shown by Wirapati et al [12], signatures derived in one cohort generally perform equally well in other cohorts (where the evaluation is usually done using direct correlations), suggesting that direct correlations can be used in this context. However, breast cancer samples derived from different cohorts represent biologically more similar entities, and therefore a signature derived from one cohort may still be largely relevant in another cohort, much more so than a cell-line derived signature or a pathway model derived from the literature.

### Constructing expression relevance networks

Given a model signature we derived a relevance correlation network across the two panels of ER+ and ER-breast tumours as follows. First, we computed Pearson correlations between every pair of genes in the model signature also present in our ER+ and ER- expression data sets. The Pearson correlation coefficients were then transformed using Fisher's transform

(1) $$y_{ij}=\frac{1}{2}\log\frac{1+{\text{c}}_{ij}}{1-{\text{c}}_{ij}}$$

where *c_ij _*is the Pearson correlation coefficient between genes *i *and *j*, and where *y_ij _*is, under the null hypothesis, normally distributed with mean zero and standard deviation $1/\sqrt{N_{s}-3}$ with *N_s _*the number of tumour samples. Standard tests for significantly non-zero *y_ij _*led to a corresponding p-value matrix. To estimate the false discovery rate (FDR) we needed to take into account the fact that gene pair correlations do not represent independent tests. Thus, we randomly permuted each gene expression profile across tumour samples (a Monte Carlo run) and selected a p-value threshold (0.0001) that yielded a negligible average FDR (on average less than 1 false positive as averaged over 1000 Monte Carlo runs). Gene pairs with correlations that passed this p-value threshold were assigned an edge in the resulting relevance expression correlation network.

### Evaluating significance and consistency of relevance networks

The significance of the relevance networks was first evaluated by comparing the average connectivity of the observed networks with those of random subsets of genes. Specifically, for each pathway in each of the ER+ and ER- subtypes we used 1000 random selections of genes from the same merged data set and recomputed the average connectivity of the resulting network. A p-value of significance was then derived as the fraction of randomisations that yielded an average connectivity larger than the observed one.

The consistency of the derived pathway networks with the prior model pathway information was evaluated as follows: given an edge in the derived network we assigned it a binary weight (1,-1) depending on whether the correlation between the two genes is positive (1) or negative (-1). This binary weight can then be compared with the corresponding weight prediction made from the model signature, namely a 1 if the two genes are either both upregulated or both downregulated in response to the oncogenic perturbation, or -1 if they are regulated in opposite directions. Thus, an edge in the network is consistent if the sign is the same as that of the model prediction. A consistency score for the observed network is obtained as the fraction of consistent edges. To evaluate the significance of the consistency score we used a randomisation approach. Specifically, for each edge in the network the binary weight was drawn from a binomial distribution with the binomial probability estimated from the merged data sets. We estimated the binomial probability of a positive weight (1) as the fraction of positive pairwise correlations among all significant pairwise correlations and was found to be 0.6 and 0.56 for the ER- and ER+ data sets, respectively. A total of 1000 randomisations were performed to derive a null distribution for the consistency score, and a p-value was computed as the fraction of randomisations with a consistency score higher than the observed one.

### Module detection in networks

Given a network of *n *genes with adjacency matrix *A_ij _*(*A_ij _*= 1 if *i *and *j *are significantly correlated/anti-correlated, otherwise *A_ij _*= 0) we were interested in identifying modules/communities in this network, defined as a partition of the network into subnetworks where the internal edge density is relatively high compared to the external one. This is analogous to finding clusters of locally significantly correlated genes, given the construction of the network. Here we used a solution to the community detection problem based on the optimization of a quality function called *modularity *proposed in [30], which allows the comparison of different partitionings of the network. Given a network partitioned into communities, being *C_i _*the community to which node *i *is assigned, the mathematical definition of modularity is expressed in terms of the adjacency matrix as

(2) $$Q=\frac{1}{2E}\sum_{i}\sum_{j}\left(A_{ij}-\frac{k_{i}k_{j}}{2E}\right)\delta (C_{i},\;C_{j}),$$

where *E *is the number of edges in the network, and *k_i _*= ∑*_j _A_ij _*refers to the degree of node *i*. The Kronecker delta function *δ*(*C_i_*, *C_j_*) takes the values, 1 if nodes *i *and *j *are in the same community, 0 otherwise.

The modularity of a given partition is then the probability of having edges falling within groups in the network minus the expected probability in an equivalent (null case) network with the same number of nodes, and edges placed at random preserving the nodes' degree. The larger the value of modularity the best the partitioning is, because more deviates from the null case. Several authors have attacked the problem proposing different optimization heuristics [30-32,47-50] since the number of different partitions grows at least exponentially with the number of nodes *n*. Here, optimization of modularity was performed using two different algorithms [32,51] and the best solution from 50 runs was used as the final partition.

### Pathway activation metrics

We initially defined two main classes of pathway activation metrics on a given gene module. One metric is based on single-gene based expression profiles for the genes in the module, while the other uses the network structure/topology of the module into account. The latter metric is motivated by the fact that the module over which pathway activity is to be estimated (MPA) does not generally constitute a clique, and therefore a score of pathway activation should take the structure of the module into account.

First, we define the single-gene based pathway activation metric. This metric is similar to the subnetwork gene expression metric used in the context of protein-interaction networks [6]. The metric for the module (MPA) of size *M *is defined as,

(3) $${\vec{s}}_{1}=\frac{1}{\sqrt{M}}\sum_{i\in MPA}\sigma_{i}{\vec{z}}_{i}$$

where ${\vec{z}}_{i}$ denotes the z-score normalised (mean zero and unit variance) expression profile of gene *i *across the tumours and *σ_i _*denotes the sign of pathway activation (from the in-vitro model signature data), i.e *σ_i _*= 1 if upregulated upon activation, *σ_i _*= -1 if downregulated. Thus, this metric, while it only takes those genes in the MPA into account, it ignores the detailed topological structure of the MPA.

To motivate the other class of pathway activation metrics, we first rewrite the single-gene based metric in terms of gene-pairs,

(4) $${\vec{s}}_{1}=\frac{1}{(M-1)\sqrt{M}}\sum_{(ij)\in P_{MPA}}f({\vec{z}}_{i},\;{\vec{z}}_{j})$$

where $f({\vec{z}}_{i},\;{\vec{z}}_{j})=\sigma_{i}{\vec{z}}_{i}+\sigma_{j}{\vec{z}}_{j}$ is an additive function of the gene expression profiles and where the summation is over all unique gene pairs (*P_MPA_*) in the MPA regardless of whether there is an edge between the two genes or not. Thus, this now directly motivates a pathway activation metric ${\vec{s}}_{2}$ that does take the structure of the MPA into account,

(5) $${\vec{s}}_{2}=\frac{1}{(M-1)\sqrt{M}}\sum_{(ij)\in P_{MPA}}A_{ij}(\sigma_{i}{\vec{z}}_{i}+\sigma_{j}{\vec{z}}_{j})$$

Thus, this metric is only computed along the edges in the MPA and gives more weight to those genes with most connections. We therefore expect the measure ${\vec{s}}_{2}$ to give a better representation of pathway activation since ${\vec{s}}_{1}$ also involves averaging over gene pairs that need not be significantly correlated despite common presence in the MPA. We have verified that such low-correlated gene pairs exist in our MPAs, and results on simulated data support the higher accuracy of ${\vec{s}}_{2}$ (data not shown).

### Boolean Cox regression models

We considered non-linear interaction Cox proportional hazards models. First, we binarised pathway activation levels into high (1) and low activity (0) using the median activity level across samples as the threshold. Let *b_i _*denote the binary version of the pathway activity level vector *p_i_*. We then considered Boolean regression models

(6) $$h(t|b_{1},\;b_{2})=h_{0}(t)e^{\beta B(b_{1},b_{2})}$$

where *h*(*t*) is the hazard function and *B*(*b*_1_, *b*_2_) denotes a Boolean operator of the variables *b*_1 _and *b*_2_. For two binary inputs, there are four distinct Boolean models $B_{1}=b_{1}\wedge b_{2},B_{2}=b_{1}\wedge b_{2}^{c},B_{3}=b_{1}^{c}\wedge b_{2},B_{4}=b_{1}^{c}\wedge b_{2}^{c}$, where *c *and ^ denote conjugation (NOT) and AND operations, respectively. These models were compared to each other and to those based on single pathways to determine if they provided better prognostic models. To evaluate whether an interaction model added prognostic value over the single pathway models, we compared the log-likelihood of the combined model

(7) $$h_{c}(t|b_{1},\;b_{2})=h_{0}(t)e^{\beta_{1}b_{i}+\beta_{2}B(b_{1},b_{2})}$$

to that of the single pathways

(8) $$\begin{array}{cc} h_{s}(t|b_{i})=h_{0}(t)e^{\beta b_{i}} & \forall i=1,2. \end{array}$$

using the likelihood ratio test (LRT) (1 degree of freedom). Specifically, if *l_c _*= log *L *denotes the log-likelihood of the combined model and *l_i _*that of the single-pathway model, we constructed the likelihood ratio test statistic as *LRT_i _*= 2 * (*l_c _*- *l_i_*), which under the null is *χ*^2^-distributed with 1 degree of freedom. Improved prognostic pairwise models are obtained by those for which either *LRT*_1 _or *LRT*_2 _is significantly larger than zero. Here we restricted to pairwise models where pathways were individually associated with prognosis and searched for pairwise combinations which further improved the prognostic model.

## Results

### Estimating pathway activation using expression network topology

The central hypothesis underlying our methodology is that only a proportion of the genes in the model signature will show an expression pattern across the clinical tumours that is consistent with their role as markers of pathway activation. To help identify those genes that are relevant from those that have inconsistent or irrelevant expression patterns we make use of a large mRNA expression data set of ER+ (785 samples) and ER- (438 samples) tumours over a common set of 6265 genes, obtained by merging seven different cohorts together ("Set1") [2,24,40-44]. These data sets were chosen because they represent large high quality data sets with the required clinical information (ER status and clinical outcome). The seven microarray expression data sets were merged over the common genes using a z-score normalisation procedure that we have validated previously [15,45]. We verified, by performing a PCA analysis on the merged data sets, that none of the top 20 PCs were correlated with the cohort of origin but instead where highly correlated with the intrinsic subtype, indicating that samples clustered significantly according to tumour subtype and not according to the original study (Additional file 2).

**Figure 1:**
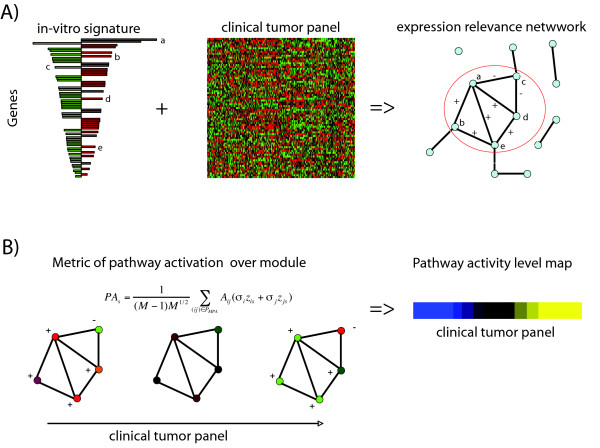
**Measuring pathway module activation**. Flowchart figure showing overall strategy used for inferring pathway module activity in clinical tumor samples from a model (perturbation) signature. **A) **A gene mRNA signature that represents a perturbed cancer cell phenotype (i.e oncogene overexpression) is combined with mRNA expression data of a large panel of clinical tumor specimens to derive an "expression relevance network" where nodes represent genes from the signature and an edge between two nodes indicates a statistically significant Pearson correlation between the two corresponding genes as measured over the clinical tumor panel. Having constructed the relevance network, the network is first pruned so that network edges that are inconsistent with prior information are removed. Signs on edges between labelled genes indicate the sign of the significant correlation between the two genes, which must be consistent with their directionality as given by the model signature. Modules defined as subnetworks with higher than average edge density are then inferred using a spectral decomposition algorithm (see Methods). **B) **For a given relatively large module, the module of pathway activation (MPA), pathway activity is then computed using a metric defined over the topology of the module. In the formula, *PA_s _*stands for the estimated pathway module activity in sample *s*, *M *is the number of genes in the module, *σ_i _*is a binary weight (1,-1) indicating the directionality of gene expression of gene *i *(1 = upregulated, -1 = downregulated), *z_is _*is the z-score normalised gene expression value in sample *s *and *A_ij _*is the adjacency matrix of the module. Effectively, this metric gives more weight to gene interactions that are supported by the data. Color and sign of nodes reflect the directionality of expression in the *in-vitro *signature (Red = upregulated &*σ *= 1, Green = downregulated &*σ *= -1). Pathway activity levels can then be shown as heatmaps (blue = high activity, yellow = low activity).

Our strategy to estimate pathway activation for a given model signature is llustrated in Figure 1 (see also Methods) and is carried out separately for ER+ and ER- disease. Briefly, the algorithm constructs a pruned relevance correlation network of the genes in the model signature across the expression tumour panel. Only genes and correlations between genes that are consistent with the prior information are allowed in the network. This strategy therefore filters out genes and gene-pairs with irrelevant or inconsistent expression patterns, while also identifying modules of high-edge density, that is, subnetworks of genes that show consistent and significantly correlated (or anticorrelated) patterns across the panel of tumours.

To further justify the need to filter out genes with inconsistent expression profiles we show that not doing so can lead to biologically inconsistent results. Using all genes in two signatures of *ERBB2 *and *EGFR *activation [1,3] to infer pathway activity in a large set of breast tumour samples and using either Spearman or Pearson correlations showed that predicted *ERBB2 *activity was not highest in the intrinsic HER2+ subtype, and similarly that *EGFR *activity was not highest in the basal subtype (Additional file 3). These inconsistencies are caused by a significant proportion of the genes in the signatures not exhibiting the expected correlations.

### Significance and consistency of expression correlation networks

We first observed that the relevance correlation networks for the model signatures contained on the order of 10% to 25% of the maximum possible number of edges (Table 1). We asked if this connectivity was higher than that of a random subset of genes. In spite of the much higher connectivity in ER+ disease, comparison to the null distribution showed that not all networks in ER+ disease where significant (Table 1). In contrast, all reasonably sized networks in ER- disease showed higher connectivity than that expected by chance (Table 1). Next, we asked if the edges of the networks, representing significant correlations or anti-correlations, were consistent with the prior information of the model signature (Table 1, Methods). Reassuringly, almost all networks showed statistically significant consistency (*P *< 0.001) with the model data indicating the potential of using such model signatures to estimate pathway activity across clinical tumour specimens (Table 1). Consistency scores however varied considerably depending on the pathway considered (50% - 100%). In view of the fact that a proportion of edges showed inconsistent patterns with the model data, these were removed to yield "pruned" correlation networks.

**Table 1:** Molecular pathways and properties of their expression networks

ER-						
**Pathway**	**nG**	**nE**	**fE**	**Pval(signif)**	**fconsE**	**Pval(consist)**

MYC	76	472	0.17	< 0.001	0.88	< 0.001
E2F3	104	793	0.15	< 0.001	0.58	< 0.001
RAS	135	1387	0.15	< 0.001	0.63	< 0.001
ERBB2	228	4223	0.16	< 0.001	0.84	< 0.001
EGFR	180	2395	0.15	< 0.001	0.66	< 0.001
AKT	41	346	0.42	< 0.001	1.00	< 0.001
IL12	17	38	0.28	< 0.001	0.95	< 0.001
IL4	11	9	0.16	0.11	1.00	< 0.001
IL2	19	22	0.13	0.19	0.73	0.009
IL13	7	6	0.29	0.01	1.00	< 0.001
INFG	40	222	0.28	< 0.001	0.90	< 0.001
TGFB	62	479	0.25	< 0.001	0.92	< 0.001

**ER+**						

**Pathway**	**nG**	**nE**	**fE**	**Pval(signif)**	**fconsE**	**Pval(consist)**

MYC	76	749	0.26	0.24	0.79	< 0.001
E2F3	104	1285	0.24	0.58	0.57	< 0.001
RAS	135	2231	0.25	0.48	0.57	< 0.001
ERBB2	228	7336	0.28	0.01	0.74	< 0.001
EGFR	180	4286	0.27	0.13	0.66	< 0.001
AKT	41	676	0.82	< 0.001	1.00	< 0.001
IL12	17	32	0.23	0.53	0.97	< 0.001
IL4	11	10	0.18	0.72	0.70	0.06
IL2	19	40	0.23	0.51	0.60	0.08
IL13	7	6	0.29	0.28	0.50	0.35
INFG	40	284	0.36	0.003	0.83	< 0.001
TGFB	62	713	0.38	< 0.001	0.86	< 0.001

Properties of the inferred relevance expression correlation networks in ER- and ER+ breast cancer. For each molecular pathway we give the number of pathway genes present in the expression matrix (nG), the number and fraction of edges (i.e significant pairwise correlations between genes) (nE & fE), the significance of the average connectivity of the network (Pval(signif)), the fraction of edges that are consistent with the prior *in-vitro *information (fconsE), the corresponding p-value of significance (Pval(consist)). P-values were estimated using 1000 permutations.

### Modular structure of molecular pathways

Next, we applied a spectral decomposition algorithm [50,51] to infer subnetworks of relatively high edge density, which we called modules (Methods). We confirmed the modularity of the networks and the presence of relatively small outlier modules in several pathways (Additional file 4). Given the smaller size of the immune response and interferon pathway gene lists, these pathways were not broken up into modules. For the larger model signatures containing several large modules, we explored if pathway activation would be dependent on the specific module. Thus, we estimated the activity for the largest modules in each pathway and asked if the activities of the individual modules were highly correlated. Interestingly, this showed that many modules within a pathway were not highly correlated and that in some cases correlations were even negative (Additional file 5). This result agrees with findings reported in [5]. In order to arrive at a single activation measure for each pathway, we therefore selected the module containing the gene undergoing the perturbation. This criterion could be used to select modules for the MYC (*MYC*), RAS (*HRAS*), ERBB2 (*ERBB2*), AKT (*AKT1*) and EGFR (*EGFR*) pathways. For the E2F3 and TGFB pathways we used *CCNE1 *and *COL3A1*, which are well known downstream targets of *E2F3 *and *TGFB*, respectively [1,38]. Given the smaller size of the immune response and interferon pathways, pathway activity estimation for these was performed on the whole network (i.e no module selection). Gene members, their interactions in the selected modules plus directionality of regulation are listed in Additional file 6. Heatmaps of all genes in the selected modules across ER+ and ER- breast cancer confirmed their significant within-module correlations and anticorrelations (Additional file 7).

### Patterns of pathway activation correlate with intrinsic subtypes

**Figure 2:**
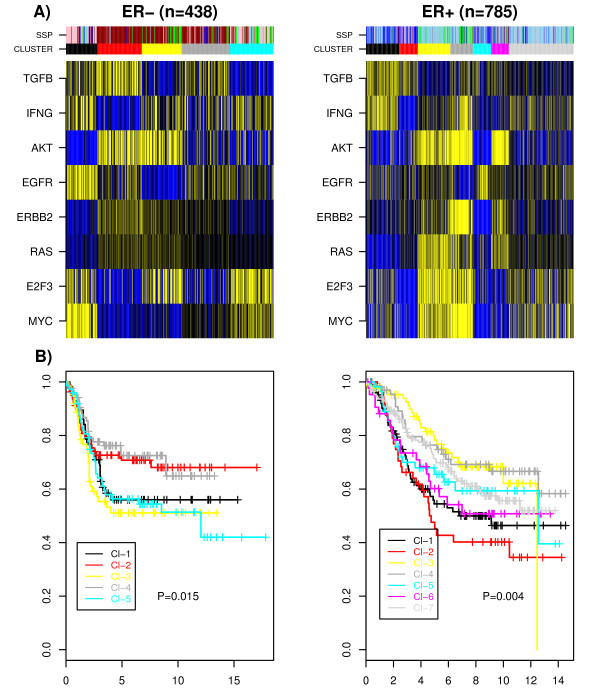
**Clustering analysis over pathway modules**. A) Heatmaps of pathway activation (blue = high relative activation, yellow = low relative activation) over the merged ER- and ER+ cohorts [2,24,40-44]. Color bars indicate the intrinsic subtype (Pink = HER2+, green = normal, dark-red = basal, skyblue = luminal A, blue = luminal B) and the cluster inferred using a variational Bayesian method [52]. B) Kaplan Meier plots for distant metastasis free survival (DMFS) for the predicted clusters in ER- and ER+ breast cancer, respectively.

The estimation of activity levels for the selected modules across clinical tumours yielded a pathway activity level matrix. Clustering was performed using a variational Bayesian mixture model [52] over the 8 largest molecular pathway modules to see if samples segregated significantly according to intrinsic subtype [46] (Figure 2A). We observed that inferred clusters mapped to intrinsic subtypes, as well as providing evidence for further heterogeneity within subtypes, confirming similar results reported in [14]. In line with the fact that intrinsic subtypes in ER+ breast cancer show differences in distant metastasis free survival (DMFS), inferred clusters also correlated significantly with outcome (Figure 2B). Importantly, we observed a significant survival difference in ER- breast cancer with those samples having overactive TGFB exhibiting worst survival (Figure 2B).

**Figure 3:**
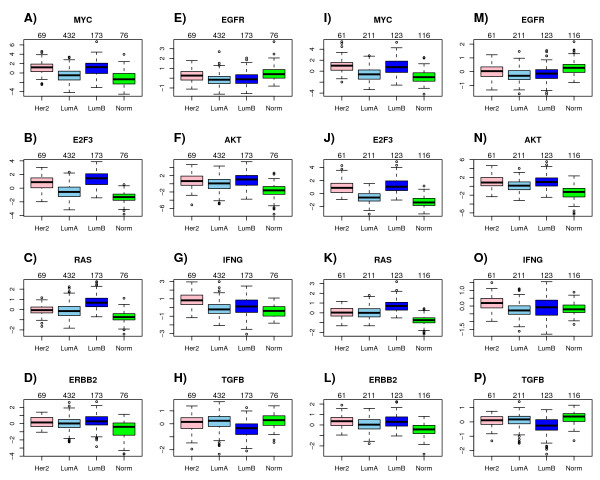
**Pathway module activation scores across intrinsic subtypes in ER+ breast cancer**. **A-H) **For the selected module in each pathway, we show boxplots of predicted pathway module activation scores across the major intrinsic subtypes within ER+ breast cancer as estimated in Set1. **I-P) **Corresponding boxplots as estimated in Set2. Number of samples in each subtype shown above corresponding boxplot. (Color Code: green = normal, skyblue = lumA, blue = lumB, pink = HER2+).

**Figure 4:**
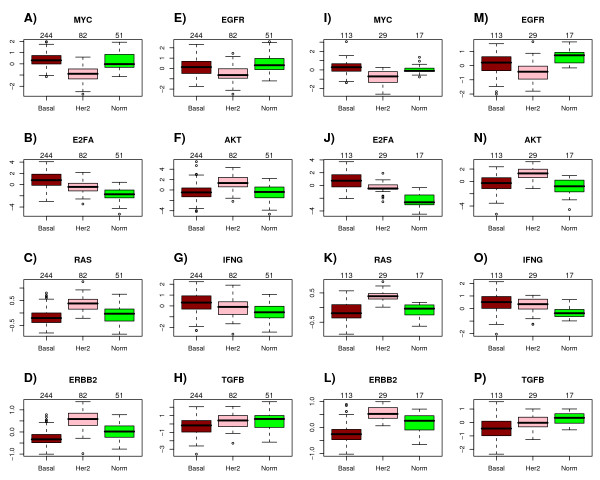
**Pathway module activation scores across intrinsic subtypes in ER- breast cancer**. **A-H) **For the selected module in each pathway, we show boxplots of predicted pathway module activation scores across the major intrinsic subtypes within ER- breast cancer as estimated in Set1. **I-P) **Corresponding boxplots as estimated in Set2. Number of samples in each subtype shown above corresponding boxplot. (Color Code: green = normal, pink = HER2+, red = basal).

From the heatmap and boxplots of pathway activity across intrinsic subtypes (Figures 3A-H &4A-H, Additional file 8) we could draw the following observations, all of which are consistent with prior knowledge:

• Activity of the ERRB2 pathway was highest in the HER2+ subtype (*P *< 10^-10^).

• Activity of the EGFR signalling pathway was highest in the basal and normal subtypes of ER- breast cancer in line with the higher levels of EGFR in these tumours [2] (*P *< 10^-8^).

• Higher activation of MYC and E2F3 pathways in luminal-B tumours compared with luminal-A, an observation consistent with many previous results associating amplification of the 8q24 locus and overexpression of cell-cycle and proliferation genes with the more aggresive luminal-B phenotype [43,53-55] (*P *< 10^-10^).

We also observed other patterns of interest that lend further support for similar results reported elsewhere:

• Higher AKT activity in the ER-/HER2+ subtype as compared to ER- basal breast cancer [2,11](*P *< 10^-10^).

• Higher HRAS activity in luminal-B tumours relative to luminal-A [2] (*P *< 10^-10^).

• Lower HRAS activity in basal tumours, with a corresponding lower expression of HRAS in basals as compared to ER-/HER2+ [2](*P *< 10^-10^).

Thus, these patterns yield insight into which molecular pathway modules are differentially activated between intrinsic subtypes.

### Pathway activation patterns are preserved in independent cohorts

In order to check the robustness of the pathway activity patterns in relation to the intrinsic subtype classification, we asked whether the identified modules showed the same pattern of variation in external independent cohorts. To this end, we collected the normalised expression data for four additional breast cancer cohorts [18,56,57] including the expression oncology (expO) data set (*http://expo.intgen.org/geo/*). This validation set (Set2) thus consisted of 657 ER+ and 173 ER- tumour samples. Pathway activity scores for the modules derived from the large training set were then evaluated in each of these test cohorts using the same metric as used in the training set and subsequently merged together. Thus, only edges significant in the training set were used to evaluate pathway activity in the validation sets. We found that the patterns of differential activation for each of the modules was highly consistent between training and validation sets, indicating that (i) our methodology for evaluating activity scores is robust, and (ii) that the identified modules may have biological significance (Figures 3I-P &4I-P). In fact, we asked how many of the predicted (i.e significant) pathway activation differences between major SSP subtypes in each ER+/ER- class were also significantly different in Set2 (Additional file 8). This showed that for ER+ and ER- disease, 92% and 81% of all pairwise significant differences in the training set were also significantly different in the validation set, with 98% and 100% of these showing the same directional change. In addition, we also observed consistency in the scale and range of activation scores for a given pathway across training and validation sets (Figures 3 &4).

### Correlations between pathway modules reveals patterns of signal transduction

**Figure 5:**
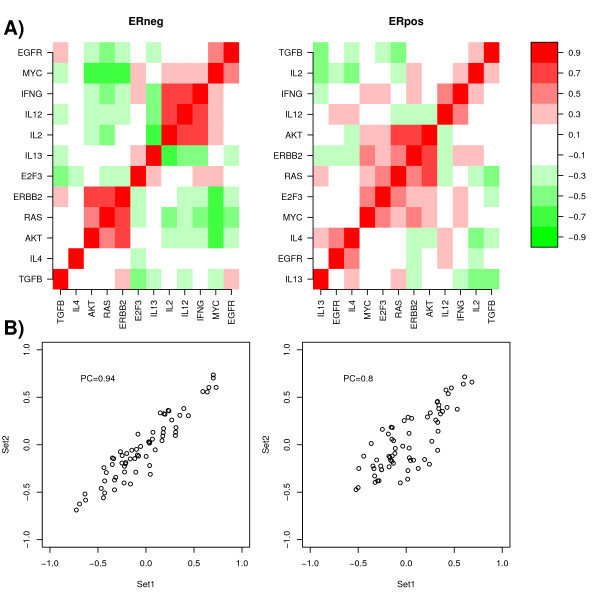
**Correlation patterns of molecular pathway modules**. **A) **Pearson correlation heatmaps between molecular pathway modules in the ER- and ER+ breast cancer (Set1), respectively (Red = high positive correlation, White = zero or insignificant correlation, Green = high negative correlation). **B) **Validation of pairwise pathway module Pearson correlations in external set (Set2). Left panel (ER-), right panel (ER+). Overall Pearson correlation (PC) between training (Set1) and validation set (Set2) is given.

Next, we investigated the correlation pattern between molecular pathway modules (Figure 5A). In both ER-and ER+ breast cancer we observed a strong correlation between the ERBB2, RAS and AKT pathways (Pearson correlation between RAS and AKT was 0.61 in ER+ and 0.59 in ER-), consistent with AKT-signalling a direct downstream target of RAS and ERBB2 [3,58,59]. Interestingly, in ER+ breast cancer these pathways were also correlated with MYC and E2F3. MYC and E2F3 pathways showed mutual strong correlations (Pearson correlations: 0.59 in ER+, 0.24 in ER-), consistent with E2F being a known transcriptional downstream target of MYC [60,61]. Another cluster was made up of immune response pathways.

Specifically, IL12, IL2 and IFNG, all involved in Th1 mediated immune response [25,26], showed strong correlations in both ER+ and ER- breast cancer, while IL13 (involved in a Th2 immune response) was generally anti-correlated to these pathways.

To evaluate the robustness of these patterns we computed the pairwise module correlations in the external cohort set (Set2) and compared these values to the ones in Set1. We observed strong agreement between the two data sets (Figure 5B).

### Pathway interactions define novel prognostic subclasses

Next, we asked if individual module activation levels were correlated with distant metastasis free survival (DMFS). Pathway module activation levels were dichotomised into high and low activity in order to help interpretability of pathway interaction terms and ease comparison between multiple and single pathway models. First, using univariate Cox-proportional hazards regression models we found that E2F3, MYC and RAS overactivation were associated with poor prognosis in ER+ breast cancer (*P *< 0.01, Table 2). In a multivariate model including all three, only E2F3, which defines a proliferation module, remained prognostic. Interestingly, while IL12 and IL2 activation showed a trend towards favourable outcome, IL13 was marginally associated with poor prognosis (Table 2). In ER- breast cancer, IL12, IL2 and IFNG were significantly associated with good prognosis, while TGFB and EGFR pathway activation were associated with poor clinical outcome (Table 2). Similar to the pattern in ER+ disease, IL13 was marginally associated with poor clinical outcome in ER- breast cancer (Table 2). We observed similar patterns of association in Set2, and in particular while IL12, IL2 and IFNG were associated with good prognosis in ER- breast cancer, IL13 correlated with poor outcome (Additional file 9).

**Table 2:** Correlations of pathway modules with outcome in breast cancer

	ER+ (n = 785)			ER- (n = 438)		
	**HR**	**95%CI**	**Pval**	**HR**	**95%CI**	**Pval**

MYC	1.5	1.16-1.95	0.002	0.82	0.58-1.16	0.27
E2F3	2.23	1.7-2.92	< 10^-8^	0.71	0.5-1	0.05
RAS	1.38	1.06-1.79	0.02	1.13	0.8-1.59	0.5
ERBB2	1.06	0.82-1.38	0.64	1.29	0.91-1.82	0.15
EGFR	0.93	0.72-1.21	0.59	1.82	1.28-2.59	< 0.001
AKT	1.29	0.99-1.67	0.06	1.49	1.05-2.11	0.02
IL12	0.88	0.68-1.15	0.35	0.52	0.36-0.74	< 0.001
IL4	0.96	0.74-1.25	0.77	0.93	0.66-1.31	0.69
IL2	0.88	0.68-1.14	0.32	0.7	0.5-1	0.05
IL13	1.28	0.98-1.66	0.07	1.29	0.91-1.82	0.15
IFNG	1.09	0.84-1.42	0.5	0.58	0.41-0.82	0.002
TGFB	0.93	0.71-1.2	0.57	1.65	1.16-2.34	0.004

For ER+ and ER- breast cancers in Set1 and for each pathway, we give the hazard ratio, 95%CI and the log-rank test P-value from a stratified Cox-proportional hazards regression model with cohorts as strata and with distant metastasis free survival (DMFS) as clinical endpoint. Pathway activation levels were divided into high/low activity levels according to values larger/lower than the median. Number of samples is given by n.

The association of high expression of genes in the Th1 immune response pathways (IL12, IL2, IFNG) with good prognosis is consistent with their putative tumor-inhibitory role [25,26]. Given that this tumor-inhibitory role could be compromised by antagonistic Th2 (IL13, IL4) and TGFB pathways [25,26], we hypothesized that tumours exhibiting simultaneous high Th1 and low TGFB activity may exhibit a better prognosis than tumors stratified by each pathway alone. To test this and to look for other pathway module interactions which may provide better prognostic stratifications, we applied logic (Boolean) Cox regression models to all pairwise combinations of pathways which were individually prognostic (Methods). For each pathway pair we identified the most predictive non-linear pathway combination and determined if it provided a better prognostic model (Methods).

**Figure 6:**
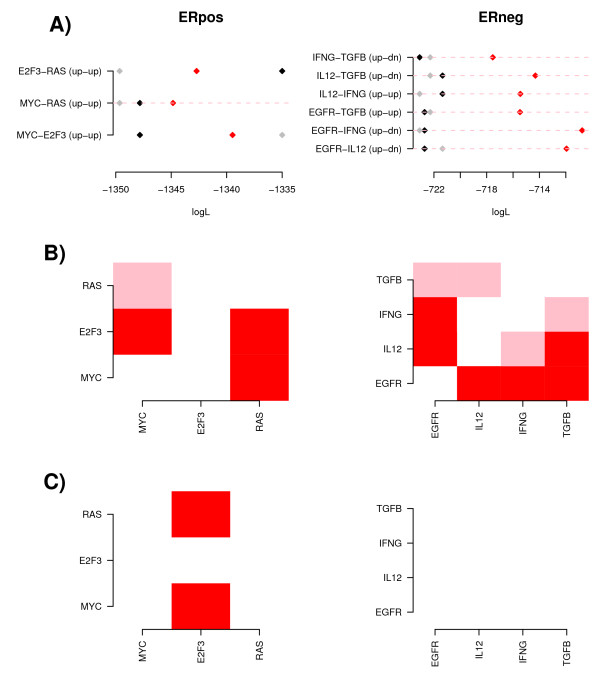
**Improved prognostic models through non-linear interactions of pathway modules**. **A) **For pathways that correlated with DMFS in ER+ and ER- breast cancer (Table 2), we consider corresponding Boolean interaction Cox regression models describing the pairwise interaction of any two pathways (the best model out of a total of 4, i.e up-up, up-down, down-up, down-down, is shown). y-axis labels the pathway interaction pair and best boolean model, x-axis denotes the log-likelihood of the corresponding model. (Black = log-likelihood of model for first pathway in pair, Grey = log-likelihood of model for second pathway in pair, Red = log-likelihood of the best Boolean interaction model, pink dashed line highlights those Boolean models with improved log-likelihoods). **B) **Heatmaps of likelihood ratio test (LRT) p-values comparing nested prognostic models. Specifically, LRT p-value for pathway *p_y _*on y-axis and pathway *p_x _*on x-axis is obtained by comparing Cox-regression models with the single pathway *p_x _*plus non-linear Boolean interaction *B*(*p_x_*, *p_y_*) as predictors against the model with only *p_x _*as predictor. **C) **As B), but LRT p-value for pathway *p_y _*on y-axis and pathway *p_x _*on x-axis is obtained by comparing Cox-regression models with the single pathway *p_x _*plus non-linear Boolean interaction *B*(*p_x_*, *p_y_*) as predictors against the model with only *B*(*p_x_*, *p_y_*) as predictor. Color codes: red (*P *< 0.01), pink (*P *< 0.05), white (*P *> 0.05). All Cox regression were stratified regression using the cohorts as strata to account for variations in the hazard rate between cohorts.

**Figure 7:**
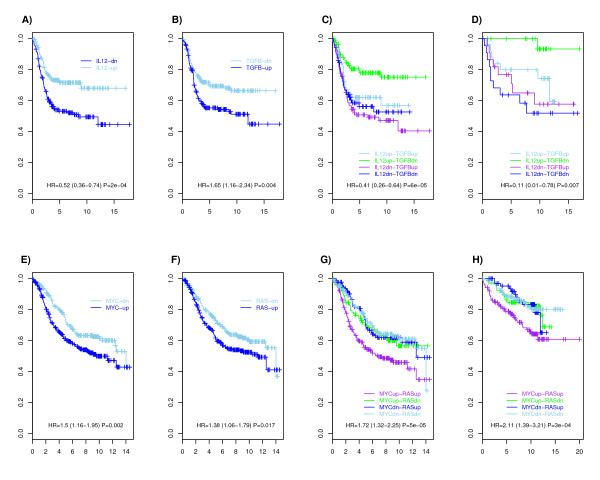
**Novel prognostic subtypes in ER- and ER+ breast cancer**. **A) & B) **Kaplan Meier DMFS curves for dichotomised pathway activity levels, for IL12 and TGFB, in ER- breast cancer (Set1). **C) **Corresponding Kaplan Meier curve for the four subtypes stratified according to up/down activity of the two pathways. Hazard ratio refers to the IL12up-TGFBdn subtype relative to the rest. **D) **Independent validation in the test cohort (Set2). **E) & F) **Kaplan Meier DMFS curves for dichotomised pathway activity levels, for MYC and RAS, in ER+ breast cancer (Set1). **G) **Corresponding Kaplan Meier curve for the four subtypes stratified according to up/down activity of the two pathways. Hazard ratio refers to the MYCup-RASup subtype relative to the rest. **H) **Independent validation in the test cohort (Set2).

In ER- breast cancer, we observed that IL12 (or IFNG) synergized with TGFB to provide a better prognostic model than either pathway considered separately (Figure 6A). Specifically, simultaneous high IL12 (or IFNG) and low TGFB activity defined a good prognosis subtype relative to all other samples (HR = 0.41 (0.26-0.64) *P *< 10^-4^) (Figure 7A). Moreover, this result held true in both basal and HER2+ subtypes (Additional file 10). Using likelihood ratio tests we verified that the non-linear interaction between IL12 (IFNG) and TGFB added prognostic value over models based on only TGFB or IL12 (Figure 6B). Conversely, the single pathway models did not improve the prognostic model provided by the non-linear interaction term (Figure 6C). We also observed that stratifying ER- samples according to high EGFR low IL12/IFNG activity provided a better prognostic model than stratifications based on the individuals pathways (Figure 6A), and Specifically that this non-linear interaction added prognostic value over the model using IL12/IFNG alone (Figure 6B). Consistent with this, simultaneous high EGFR low IL12/IFNG activity defined a subtype of poor prognosis (HR = 2.43 (1.71-3.44) *P *< 10^-6^, Additional file 11).

In ER+ breast cancer we observed that high MYC synergized with high RAS activation to provide a better prognostic stratification than either high MYC or high RAS alone (Figure 6A), and that this non-linear MYC-RAS interaction added prognostic value over the single-pathway models (Figure 6B). In contrast, adding single MYC or RAS module activities to the MYC-RAS interaction model did not improve the prognostic model (Figure 6C). We verified using Kaplan Meier curves that simultaneous high MYC and high RAS defined a subtype of poor clinical outcome (Figure 7B). However, neither of these pathways nor their interaction provided a better prognostic model than that provided by the E2F3 pathway (Figure 6).

In order to validate these findings, we dichotomised the pathway activation levels of IL12, TGFB, EGFR, RAS and MYC pathways in the ER- and ER+ samples of the test set (Set2), and first evaluated if the four subgroups, stratified according to high/low activity of the two pathways, showed differences in clinical outcome. Once again we observed that combined high IL12 low TGFB defined a good prognosis subtype in ER- breast cancer (HR = 0.11 (0.01-0.78) *P *= 0.007, Figure 7C) and that the stratification based on the combined activity levels provided a better stratification than that based on the individual pathways (Additional file 12). Similarly, simultaneous high RAS high MYC activity defined a poor prognosis subtype in ER+ disease (HR = 2.11 (1.39-3.21) *P *< 0.001, Figure 7D) and provided a better stratification than the model based on the individual pathways (Additional file 12). Although only marginally significant, high EGFR low IL12 activity displayed the same trend as in Set1, conferring poor prognosis in ER- breast cancer (HR = 1.86 (0.83-4.17) *P *= 0.12, Additional file 11).

## Discussion

We have shown that model signatures exhibit bulk tumour gene expression patterns that are generally highly consistent with the information contained in the model (Table 1). In agreement with [5] we also found that model signatures break up into distinct modules, in some cases exhibiting widely different activity patterns, supporting the view that pathway activity estimation ought to be performed after a module detection step. The modularity of the model signatures may reflect differences in cellular context (e.g comparing *in-vitro *culture to *in-vivo *conditions), the plethora of genomic abnormalities underlying any given tumour, and also inherent complex cross-talk between molecular pathways. To simplify the analysis, for those model signatures exhibiting high modularity we selected the module containing the gene undergoing the perturbation. Thus, the activation levels we report are for a specific module within the pathway and therefore may not necessarily reflect the overall pathway activation level, or provide the best estimate of pathway activation. The latter task is a complex endeavour that we hope to address in the near future using the imminent large scale multidimensional breast cancer array data sets.

However, by relating the predicted module activity patterns to the existing intrinsic subtype classification [46], we showed that the activation patterns of our inferred modules were highly preserved in independent test sets (Figures 3 &4), which not only demonstrates the robustness of our proposed method, but also shows that the pathway modules we have identified are of biological significance and that they may be used to provide an alternative clinically more relevant molecular classification of breast cancer.

Using our approach we also rediscovered known relations between molecular pathways. For example, we observed strong correlations between MYC and E2F3 pathways, consistent with E2F3 a direct downstream target of MYC (MYC→ E2F3), as well as strong correlations between ERBB2, RAS and AKT, consistent with the known signalling cascade ERBB2→RAS→AKT [58-61]. We verified that these correlations could not be explained by an overlap in the genes, as the modules exhibited minimal overlap.

**Figure 8:**
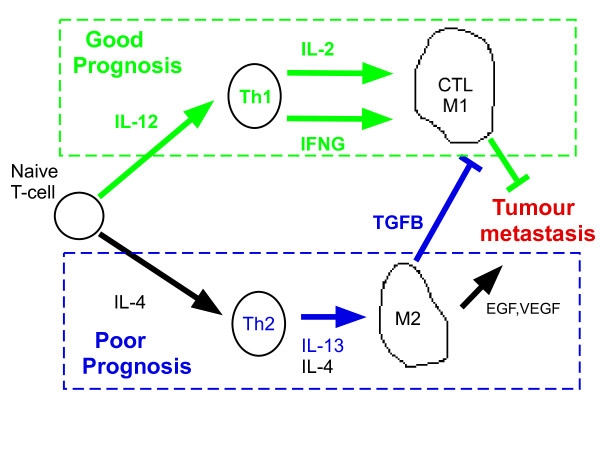
**Proposed model of how immune response pathways affect clinical outcome in ER- breast cancer**. Figure adapted from [26]. Hypothetical model in which the balance of cytokines in the breast tumour microenvironment determines the relative strength of Th1 and Th2 differentiation. Stronger activation of a Th1 immune response leads to increased production of IL2 and IFNG which mediate formation of M1 macrophages and cytotoxic killer cells, which is tumour inhibitory [26]. Correspondingly we observe that genes that are upregulated in these pathways are associated with good prognosis (DMFS) in ER- breast cancer (significant associations shown in green). Conversely, stronger activation of a Th2 immune response leads to production of IL13 and TGFB cytokines through an M2 macrophage polarization program. The cytokine TGFB is known to suppress the tumour inhibitory role of Th1 [26]. Correspondingly, we observe that genes that are upregulated in these pathways confer poor prognosis (DMFS) (significant associations shown in blue). Genes implicated in the Th1 and Th2 pathways were generally anticorrelated, indicative of an unbalanced differentiation program. It follows from this model that simultaneous high Th1 (IL2, IL12, IFNG) and low TGFB would confer better prognosis than either high Th1 or low TGFB alone, in agreement with our observations.

Interestingly, we also observed correlations between pathways (IL12, IFNG, IL2) involved in Th1 mediated immune response, as well as correlations between pathways (IL13, TGFB) that act via Th2 immune responses to putatively suppress the tumor inhibitory role of Th1 pathways (Figure 5) [25,26]. Moreover, Th1 and Th2 (IL13) pathways were generally anti-correlated and while Th1 activation was clearly associated with good prognosis, Th2 (IL13) and TGFB activation were associated with poor prognosis (Table 2 & Additional file 9). Thus, it is tentative to speculate that the balance of Th1 and Th2 differentiation pathways in the tumour microenvironment is a determinant of distant metastasis in ER- breast cancer (Figure 8). Supporting this model, we observed that ER- tumours with simultaneous low TGFB and high IL12 activity had significantly better outcome and that stratification based on the combination of these two pathways provided a better prognostic classification than those based on single pathways (Figures 6 &7). Importantly, these results were validated in an independent cohort and in both the ER-/basal and ER-/HER2+ subtypes (Additional file 10). We also verified that Th1 (IL12, IFNG) and Th2 (TGFB) modules retained the same prognostic power in multivariate models including E2F3, itself strongly prognostic in ER+ breast cancer but only marginally so in ER negatives. Since E2F3 is a proliferation module, this demonstrates once again that in ER- breast cancer, immune response pathways play a much more prominent prognostic role than proliferation.

We should point out that many of the prognostic associations we report here would only be marginal under a Bonferroni corrected threshold of (0.05/24 ~ 0.002). On the other hand a Bonferroni threshold is very stringent (probability of one false positive is 0.05) and is known to lead to a high false negative rate. Given the small number of prognostic tests being carried out (24 independent tests in total) and the clear skew towards small P-values (Kolmogorov-Smirnov test *P *= 0.001) suggests that these prognostic P-values do not derive from a uniform distribution, an indication that most of the associations reported here are unlikely to be false positives.

We also observed strong correlations of T-cell helper-1 pathways (IL12, IFNG, IL2) with the genes overexpressed in the prognostic immune-response (IR) module (*C1QA, LY9, HLA-F, TNFRSF17*) [15], a strong correlation between TGFB and the gene underexpressed in the IR-module (SPP1), as well as a strong anti-correlation between (*C1QA, LY9, HLA-F, TNFRSF17*) and IL13, which mediates T-cell helper-2 immune responses (Additional file 13). Thus, it is likely that the IR-module we identified previously [15] reflects the combined high and low activation of Th1 and TGFB pathways. Thus, given the observed synergy of these two pathways, it is tentative to suggest further that simultaneous modulation of Th1 and TGFB may be a better treatment strategy than targeting just one pathway, and further supports the rationale for combinational therapies targeting multiple pathways.

More generally, and using likelihood ratio tests, we observed that prognostic models based on module interactions gave better prognostic stratifications of samples than those based on single pathways, supporting the value of such models. Thus, in addition to the (IL12, TGFB) interaction, we observed that high EGFR and low IL12 activity provided a better prognostic model in ER- breast cancer, while simultaneous high MYC high RAS activity defined a better prognostic model in ER+ breast cancer. However, in the (MYC, RAS) case we observed that this interaction model was not better than that based on E2F3 alone, which was the strongest predictor of prognosis in ER+ disease, reflecting the well-established prognostic role of cell-proliferation genes [42]. Biologically, this makes sense because E2F3 acts downstream from both MYC and RAS, and therefore high E2F3 activity may reflect activating mutations in genes other than MYC and RAS.

## Conclusions

In summary, this work has applied a novel strategy for estimating pathway module activity levels in clinical tumours. Using this method we have shown that activation patterns of oncogenic and immune response pathway modules synergize to provide an improved prognostic classification of ER- breast cancer, further supporting the rationale for combinational therapies.

## Abbreviations

ER+: (ER positive breast cancer); ER-: (ER negative breast cancer); (HRAS): Ha-Ras1 proto-oncoprotein; (E2F3): E2F transcription factor 3; (MYC): c-myc proto-oncogene protein; (EGFR): epidermal growth factor receptor; (ERBB2): c-erb B2/neu protein; (AKT): AKT1 kinase; (IFNG): interferon-gamma; (TGFB): transforming growth factor beta-1; (IL-2,4,12,13): interleukin-2,4,12,13.

## Competing interests

The authors declare that they have no competing interests.

## Authors' contributions

AET designed the study, performed the statistical analysis and wrote the manuscript. SG and AA helped with some aspects of the statistical analysis. DEA, MS and MG contributed data. CC contributed to the writing of the manuscript. All authors read and approved the final manuscript.

## Pre-publication history

The pre-publication history for this paper can be accessed here:

http://www.biomedcentral.com/1471-2407/10/604/prepub

## Supplementary Material

Additional file 1 **Model signatures**. Model signatures used in manuscript. We provide the official gene symbol and the expected change in gene expression in response to pathway activation. Click here for file

Additional file 2 **Validation of merging algorithm**. Hierarchical clustering of merged ER+ and ER- data sets together with the distribution of intrinsic subtypes (SSP) and the cohort of origin (COHORT). For the top 20 principal components from a PCA analysis we plot the -log10(p-values) of association of the components with the SSP subtype (red) and cohort of origin (black). Green line marks the -log10(0.05) threshold. Click here for file

Additional file 3 **Direct correlation activity estimation**. Predicted *ERBB2 *and *EGFR *pathway activities based on Pearson or Spearman correlations of the signatures of *ERBB2 *and *EGFR *pathway activation from Bild et al [1] in our breast cancer data sets Set1 and Set2 combined. Pathway activation was estimated on a per-sample basis using all available genes present on the array in question. Spearman or Pearson correlations are shown across the intrinsic subtypes. Click here for file

Additional file 4 **Modularity of pathways**. Barplots showing the number (x-axis indexes the module) and sizes (y-axis) of the inferred modules for selected pathways in ER- and ER+ breast cancer, illustrating the modularity structure of pathways. Click here for file

Additional file 5 **Intra-pathway module correlations**. **A)& C) **Pearson correlations between module activation levels within molecular pathways. Only pathways with at least two modules of size larger or equal than 10 genes were selected. **A) **ER- breast cancer. **C) **ER+ breast cancer. B)Heatmap of pathway activity levels of the four predicted modules of the E2F3 pathway in ER- breast cancer. D)Heatmap of pathway activity levels of the four predicted modules of the RAS pathway in ER+ breast cancer. (Blue = high activation, yellow = low activation). Click here for file

Additional file 6 **Inferred selected modules**. Inferred selected modules within molecular pathways and for ER+ and ER- breast cancer. Each row gives the interaction and directionality of expression of each gene. Click here for file

Additional file 7 **Heatmap of module genes**. Heatmaps of gene expression (red = high, green = low) of the genes in selected modules. **A) **ER- breast cancer. **B) **ER+ breast cancer. SSP = simple sample predictor intrinsic subtype (red = basal, skyblue = lumA, blue = lumB, green = normal, pink = HER2). PATHW labels pathway, UP/DOWN labels if gene is up (black) or down (white) regulated. Grey denotes genes that are part of multiple pathways. Click here for file

Additional file 8 **Pathway activation and SSP subtypes**. For the ER+ and ER- breast cancer training (Set1) and validation (Set2) sets, we provide a table listing the sign and p-values of the Wilcoxon rank sum test for each pairwise comparison of pathway activity levels between intrinsic subtypes and for each molecular pathway. Click here for file

Additional file 9 **Clinical outcome in Set2**. For ER+ and ER- breast cancers in Set2 and for each pathway, we give the hazard ratio, 95%CI and the log-rank test P-value from a stratified Cox-proportional hazards regression model with cohorts as strata and with distant metastasis free survival (DMFS) as clinical endpoint. Pathway activation levels were divided into high/low activity levels according to values larger/lower than the median. Number of samples is given by n. Click here for file

Additional file 10 **KM-curves for IL12 and TGFB in ER- subtypes**. Kaplan Meier DMFS curves for the four subtypes stratified according to up/down activity of the IL12 and TGFB pathways. Hazard ratio refers to the IL12up-TGFBdn subtype relative to the rest. 95% confidence intervals and log-rank test P-values are given. **A) **ER- basal samples in Set1. **B) **ER- HER2+ samples in Set1. Click here for file

Additional file 11 **KM curves for IL12 and EGFR0**. **A) & B) **Kaplan Meier DMFS curves for dichotomised pathway activity levels, for IL12 and EGFR in ER- breast cancer (Set1), respectively. **C) **Kaplan Meier DMFS curves for the four subtypes stratified according to up/down activity of the IL12 and EGFR pathways in Set1. Hazard ratio refers to the IL12dn-EGFRup subtype relative to the rest. 95% confidence intervals and log-rank test P-values are given. **D) **As C) but in Set2. Click here for file

Additional file 12 **Synergy outcome maps in Set 2**. Validation of module interaction prognostic models in Set2. **A) **For the pathway modules that correlated with DMFS in the ER positive and negative breast cancer training sets and the corresponding best Boolean interaction regression model, we evaluate the association with prognosis in the validation Set2. x-axis denotes the log-likelihood of the corresponding model. (Black = log-likelihood of model for first pathway in pair, Grey = log-likelihood of model for second pathway in pair, Red = log-likelihood of the best Boolean interaction model as determined from training Set1, pink dashed line highlights those Boolean models with improved log-likelihoods). **B) **Heatmaps of likelihood ratio test (LRT) p-values comparing nested prognostic models in Set2. Specifically LRT p-value for pathway *p_y _*on y-axis and pathway *p_x _*on x-axis is obtained by comparing Cox-regression models with the single pathway *p_x _*plus non-linear Boolean interaction *B*(*p_x_*, *p_y_*) as predictors against the model with only *p_x _*as predictor. **C) **As B), but LRT p-value for pathway *p_y _*on y-axis and pathway *p_x _*on x-axis is obtained by comparing Cox-regression models with the single pathway *p_x _*plus non-linear Boolean interaction *B*(*p_x_*, *p_y_*) as predictors against the model with only *B*(*p_x_*, *p_y_*) as predictor. Color codes: red (*P *< 0.01), pink (*P *< 0.05), white (*P *> 0.05). Click here for file

Additional file 13 **Relation of pathway modules to IR-module**. Correlation heatmaps between activation levels of immune response related pathways and expression levels of the prognostic immune-response (IR) module of [15] in ER positive and ER negative breast cancer (Red = high correlation, White = zero or insignificant correlation, Green = high anti-correlation). Of the seven genes in the IR-module, five were present in Set1. Click here for file
