# Supplementary figures and images for: Improved prognostic classification of breast cancer defined by antagonistic activation patterns of immune response pathway modules
Source: BMC Cancer. 2010 Nov 4;10:604. doi: 10.1186/1471-2407-10-604 (PMC2991308; doi:10.1186/1471-2407-10-604)

ERpos

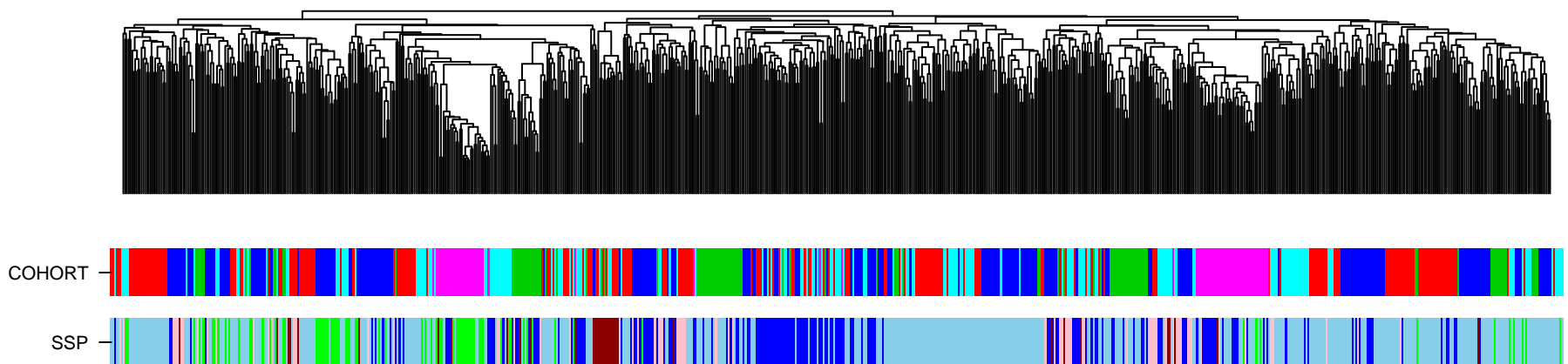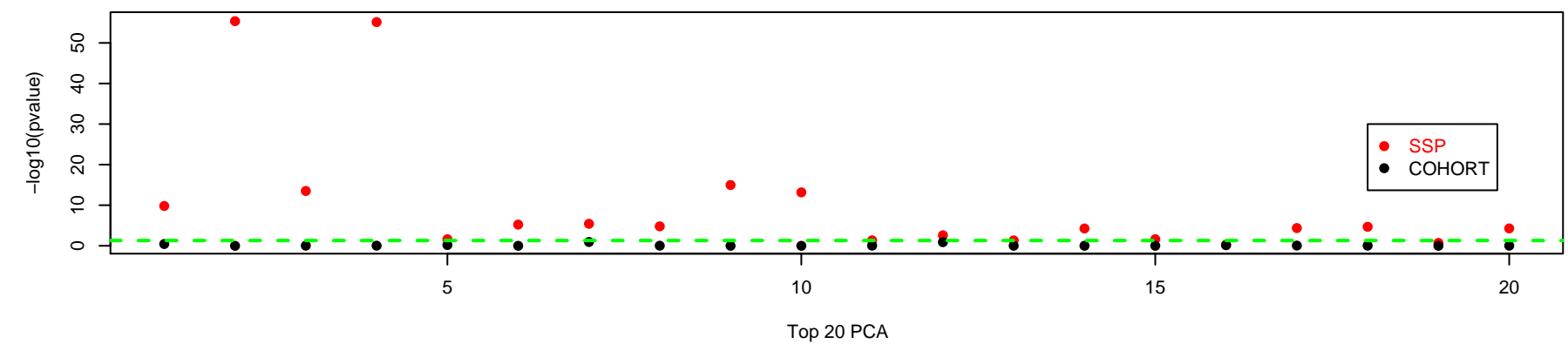

ERneg

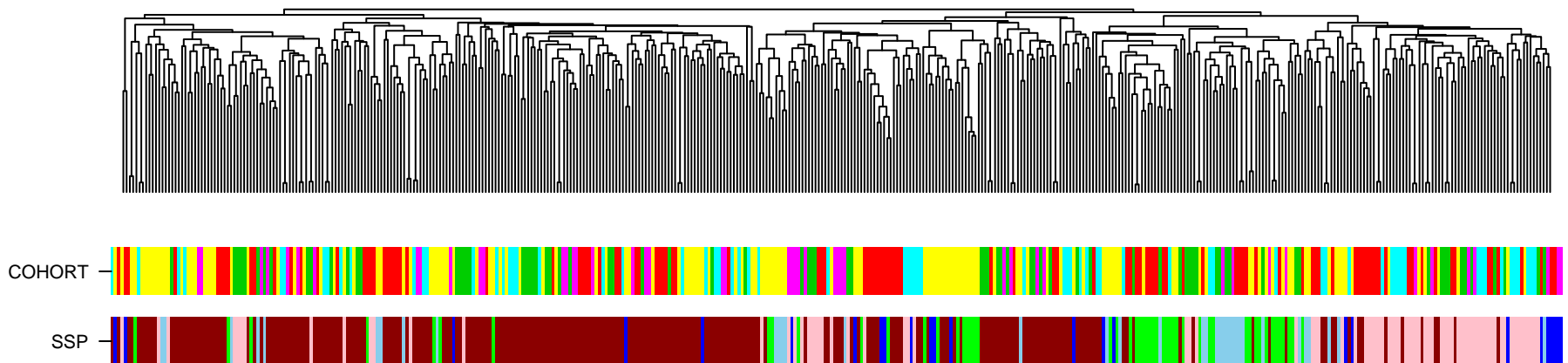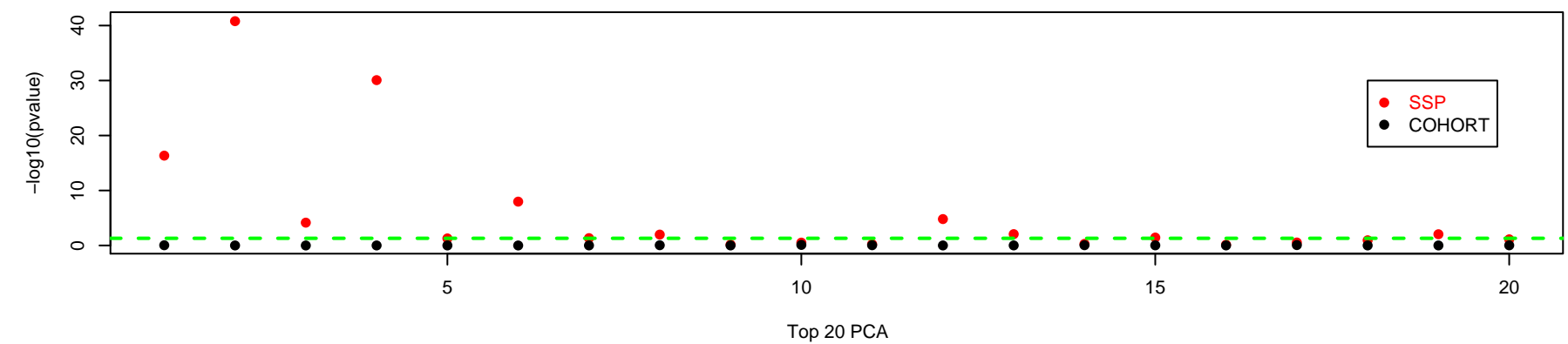

Supplement: Additional file 2 — Validation of merging algorithm. Hierarchical clustering of merged ER+ and ER- data sets together with the distribution of intrinsic subtypes (SSP) and the cohort of origin (COHORT). For the top 20 principal components from a PCA analysis we plot the -log10(p-values) of association of the components with the SSP subtype (red) and cohort of origin (black). Green line marks the -log10(0.05) threshold. [file 1471-2407-10-604-S2.PDF]

**ERBB2 –spearman**

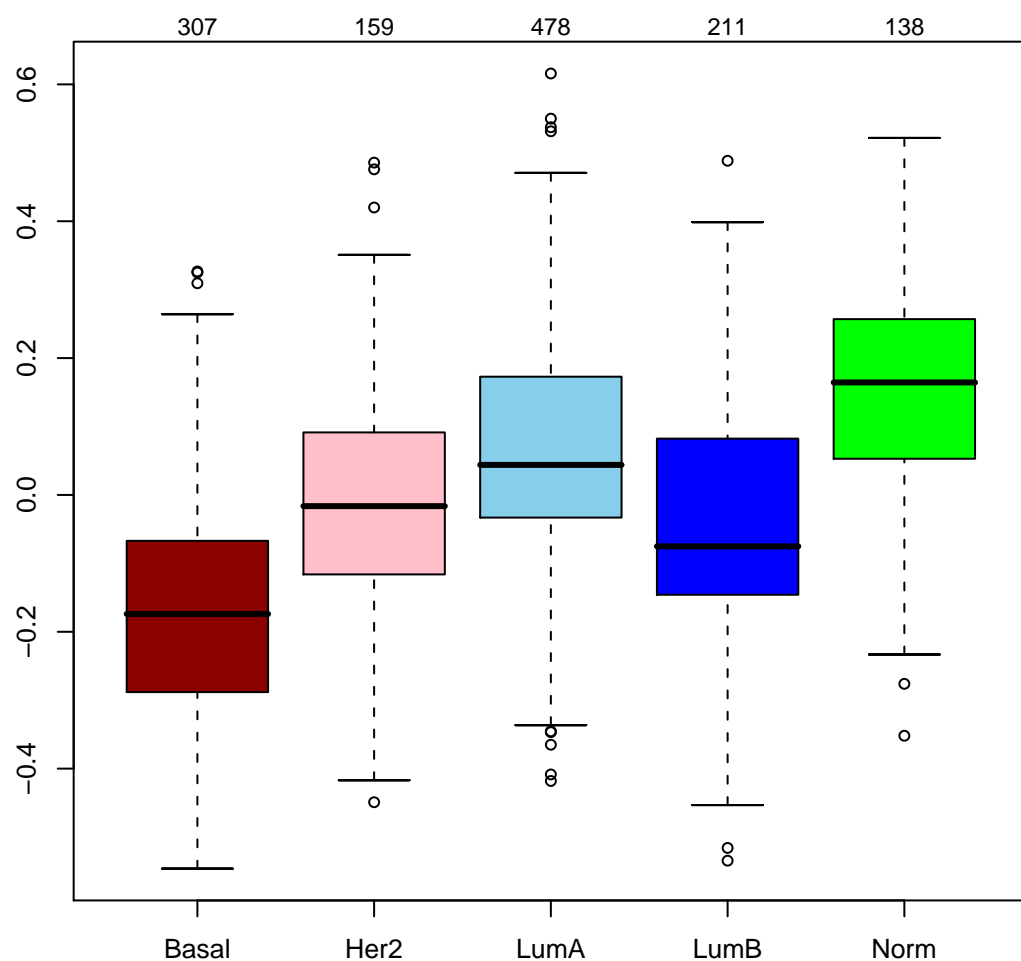

**EGFR –spearman**

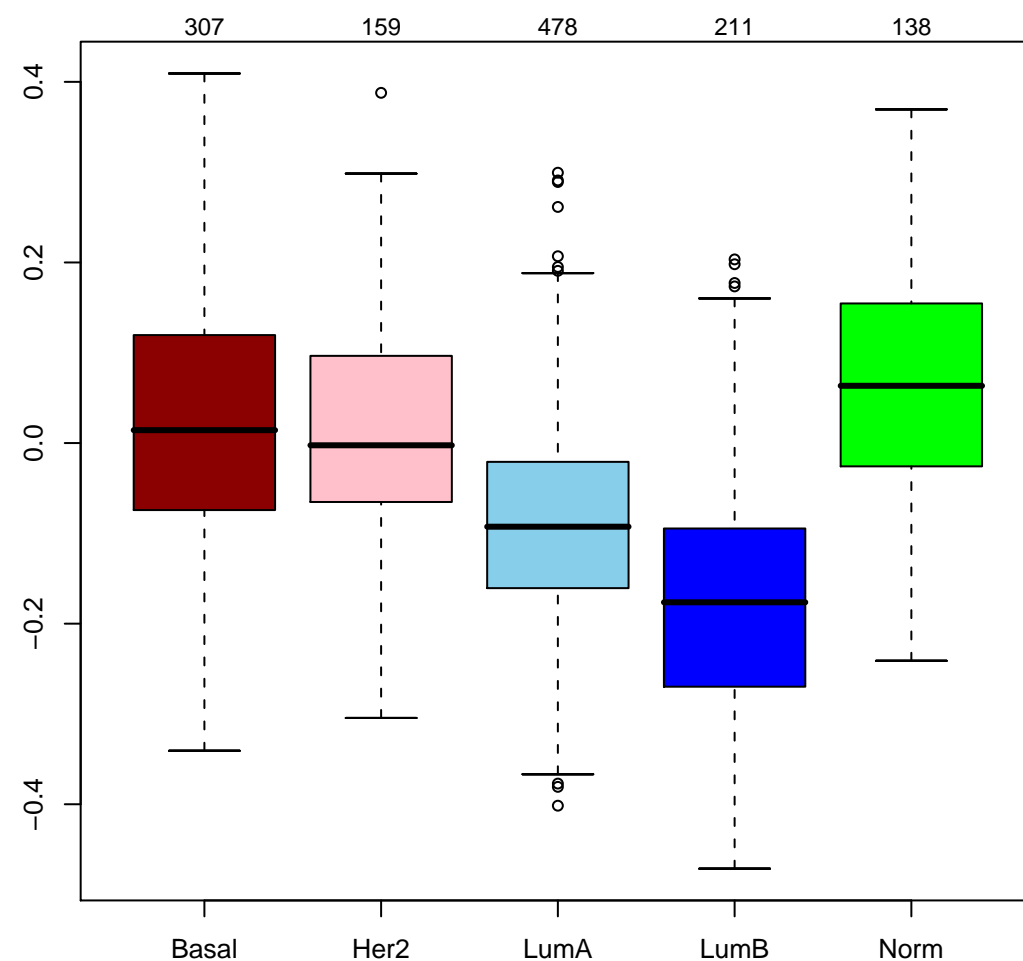

**ERBB2 –pearson**

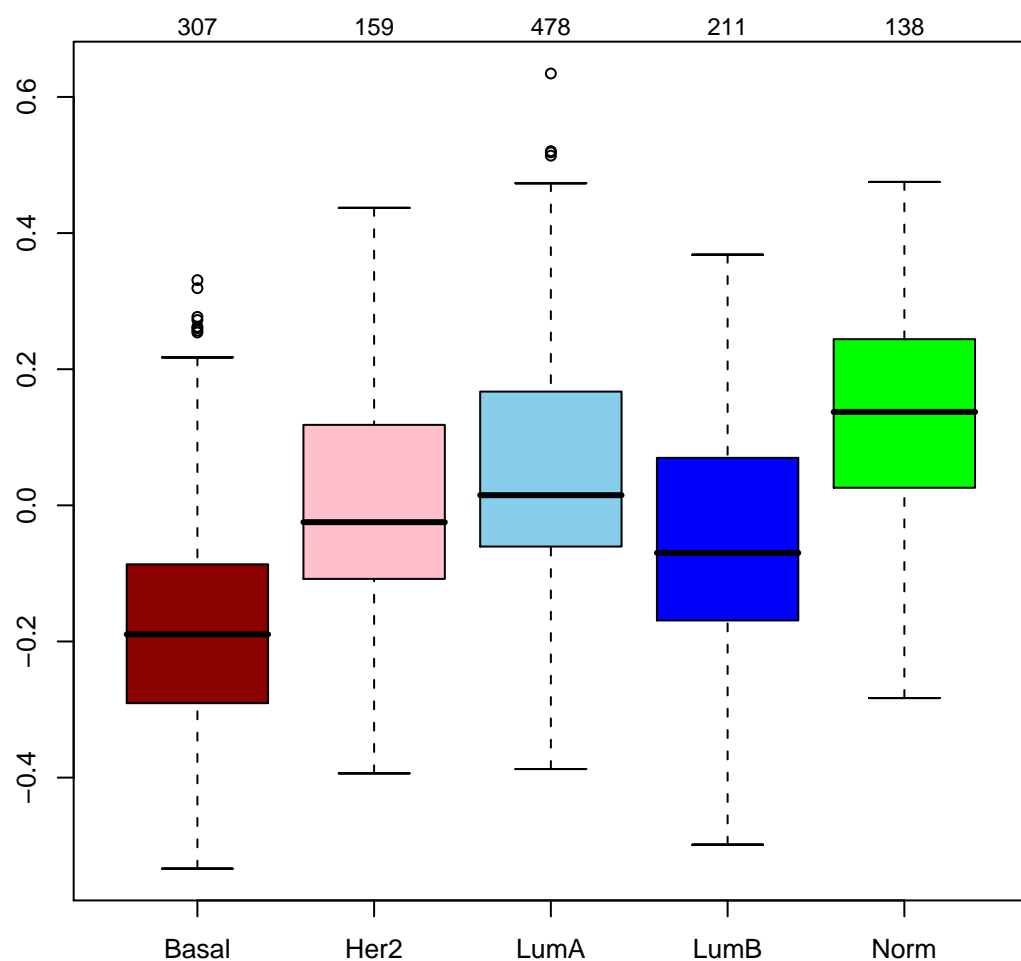

**EGFR –pearson**

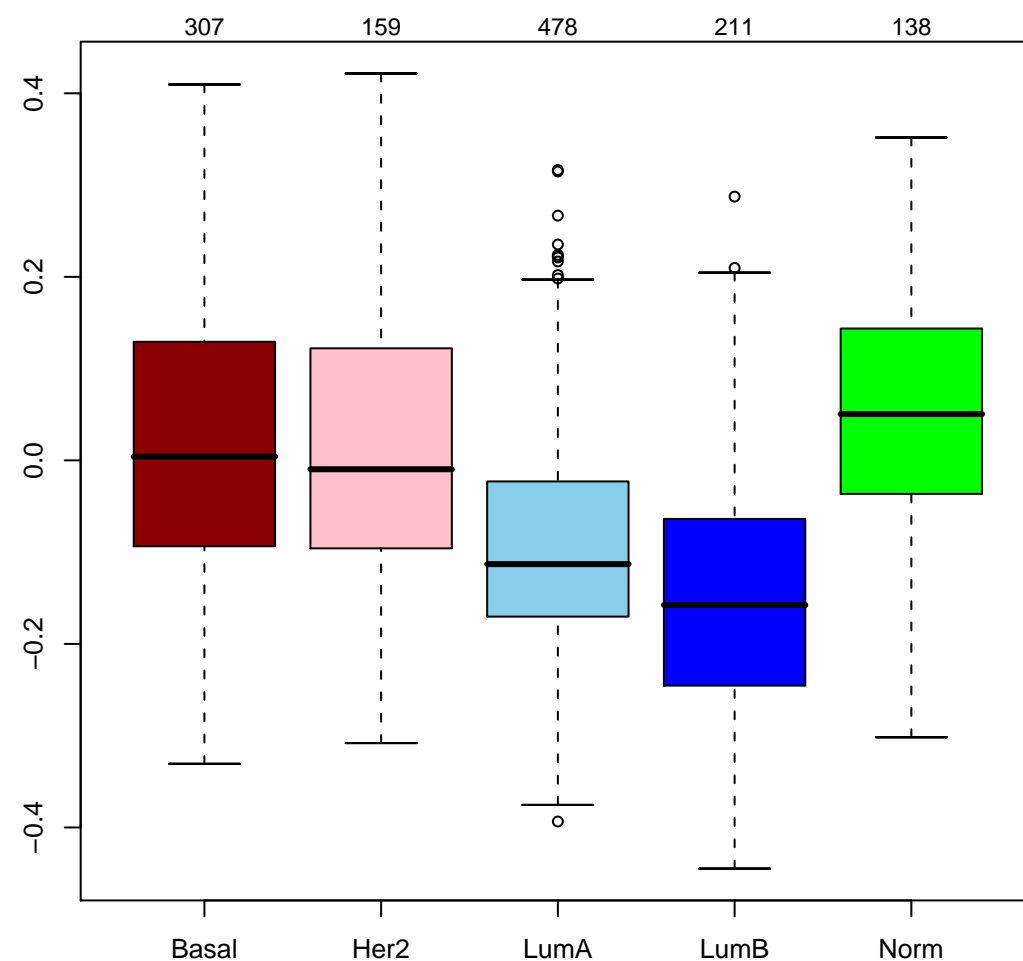

Supplement: Additional file 3 — Direct correlation activity estimation. Predicted ERBB2 and EGFR pathway activities based on Pearson or Spearman correlations of the signatures of ERBB2 and EGFR pathway activation from Bild et al [1] in our breast cancer data sets Set1 and Set2 combined. Pathway activation was estimated on a per-sample basis using all available genes present on the array in question. Spearman or Pearson correlations are shown across the intrinsic subtypes. [file 1471-2407-10-604-S3.PDF]

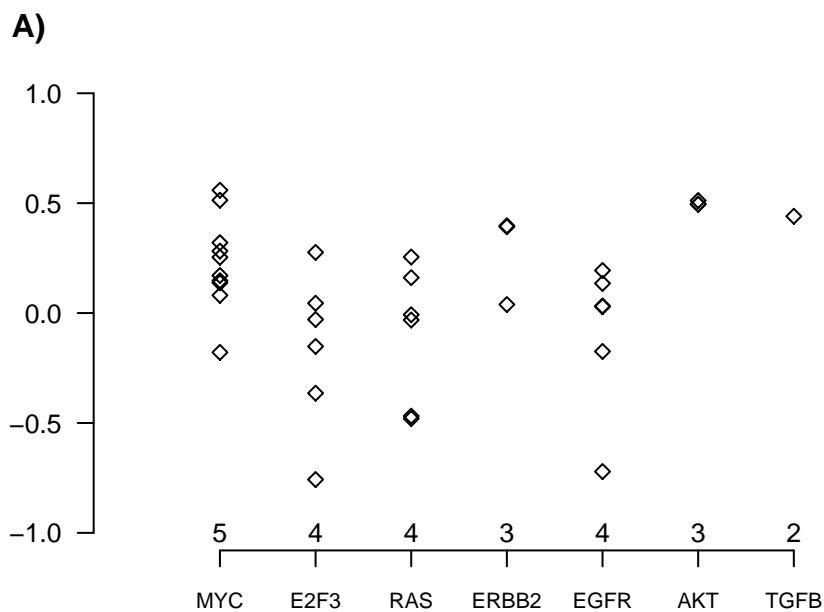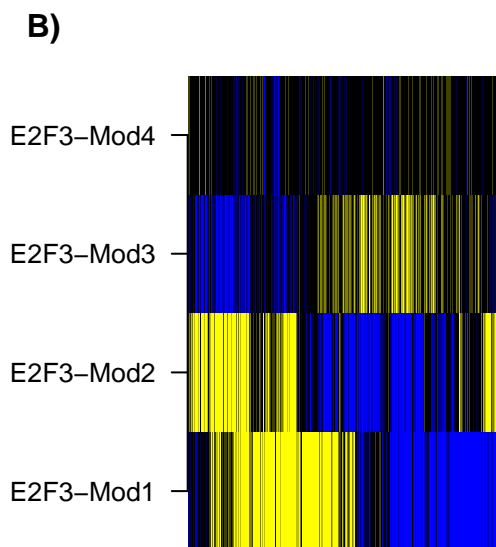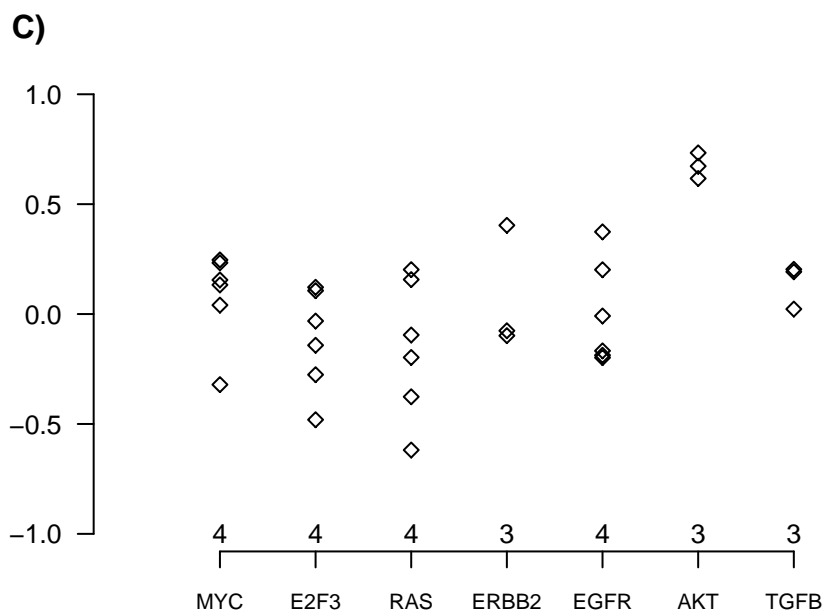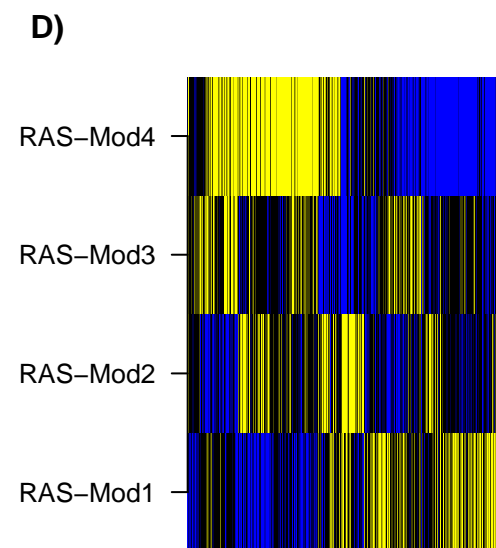

Supplement: Additional file 5 — Intra-pathway module correlations. A)& C) Pearson correlations between module activation levels within molecular pathways. Only pathways with at least two modules of size larger or equal than 10 genes were selected. A) ER- breast cancer. C) ER+ breast cancer. B)Heatmap of pathway activity levels of the four predicted modules of the E2F3 pathway in ER- breast cancer. D)Heatmap of pathway activity levels of the four predicted modules of the RAS pathway in ER+ breast cancer. (Blue = high activation, yellow = low activation). [file 1471-2407-10-604-S5.PDF]

## A) ERneg

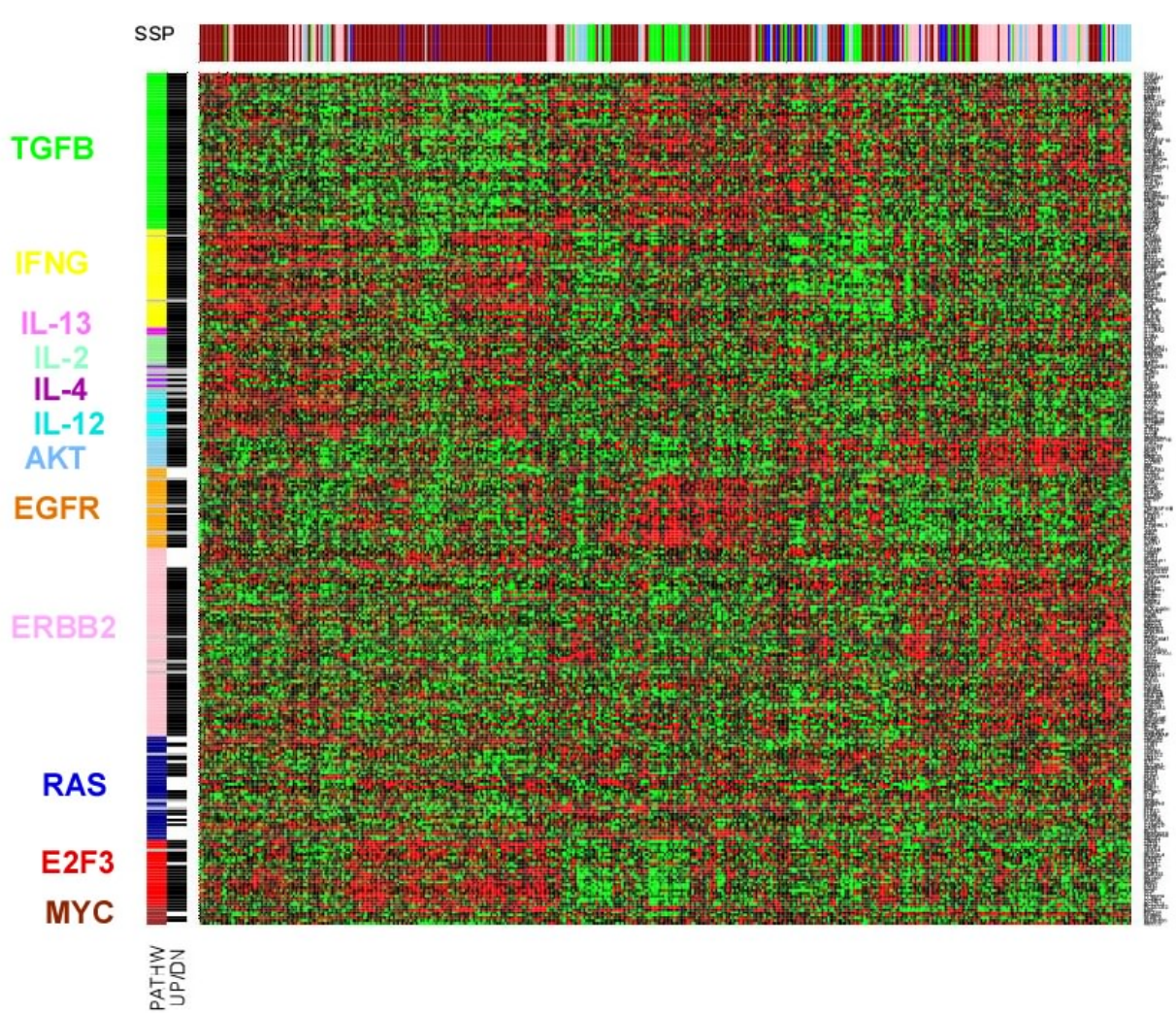

## B) ERpos

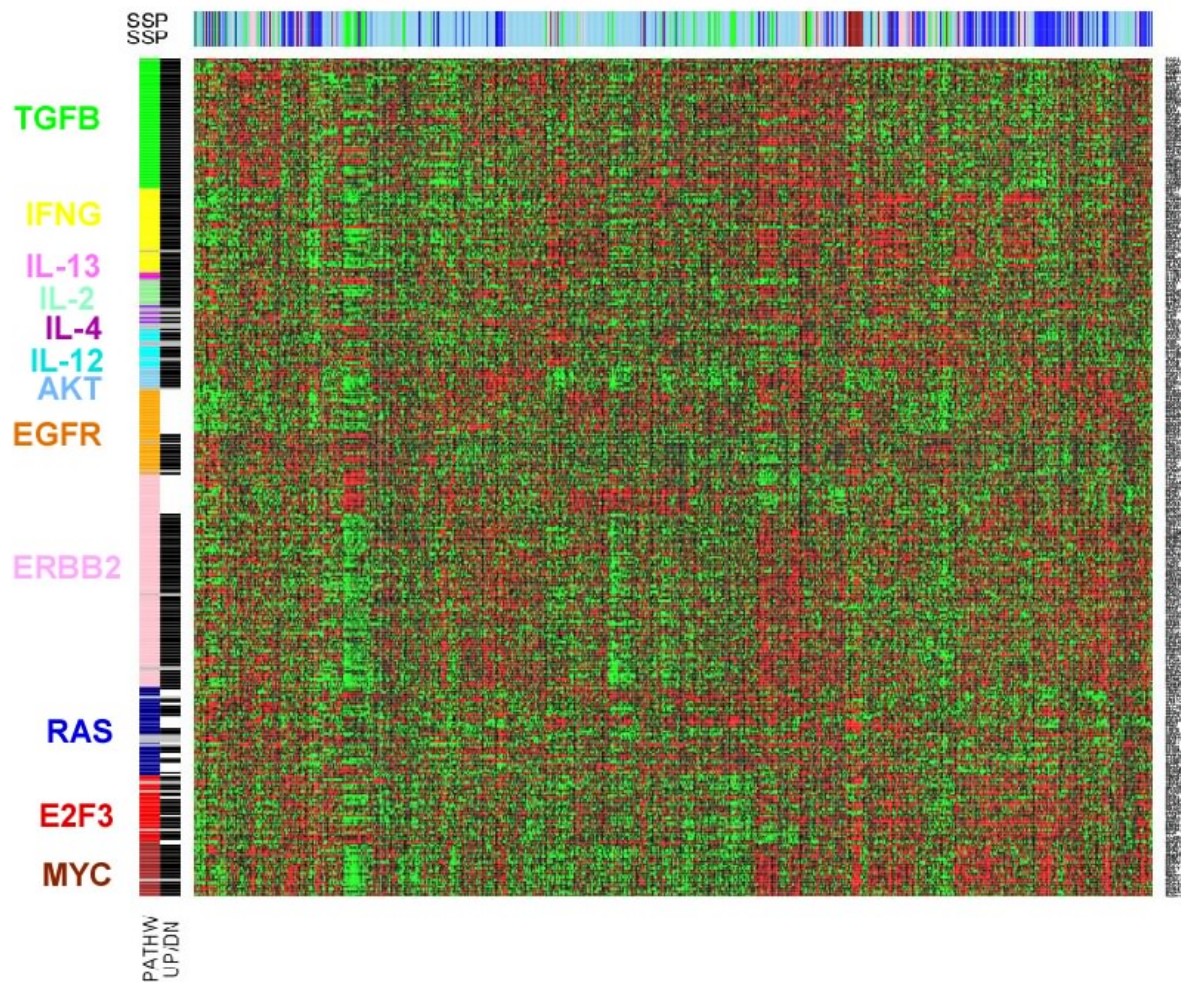

Supplement: Additional file 7 — Heatmap of module genes. Heatmaps of gene expression (red = high, green = low) of the genes in selected modules. A) ER- breast cancer. B) ER+ breast cancer. SSP = simple sample predictor intrinsic subtype (red = basal, skyblue = lumA, blue = lumB, green = normal, pink = HER2). PATHW labels pathway, UP/DOWN labels if gene is up (black) or down (white) regulated. Grey denotes genes that are part of multiple pathways. [file 1471-2407-10-604-S7.PDF]

**A) Basal (n=244)**

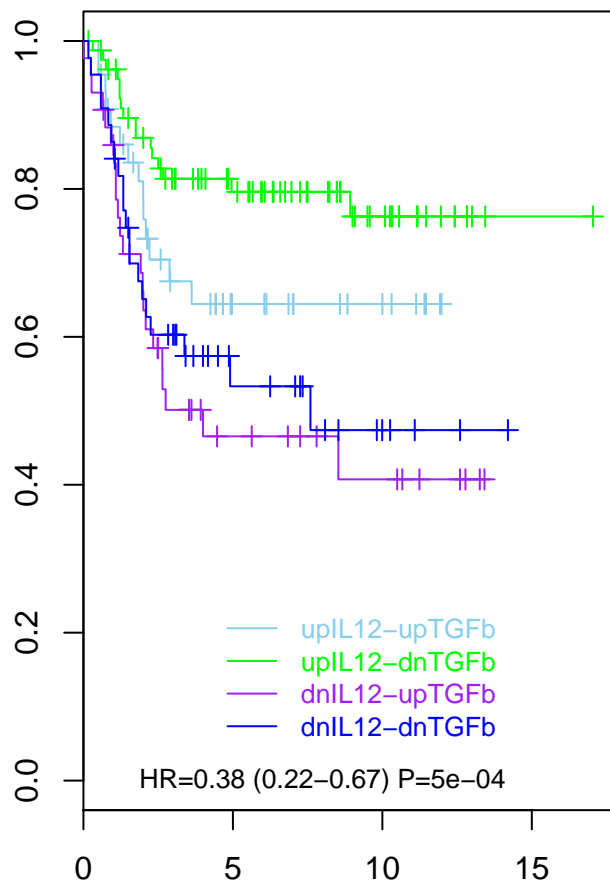

**B) HER2+ (n=82)**

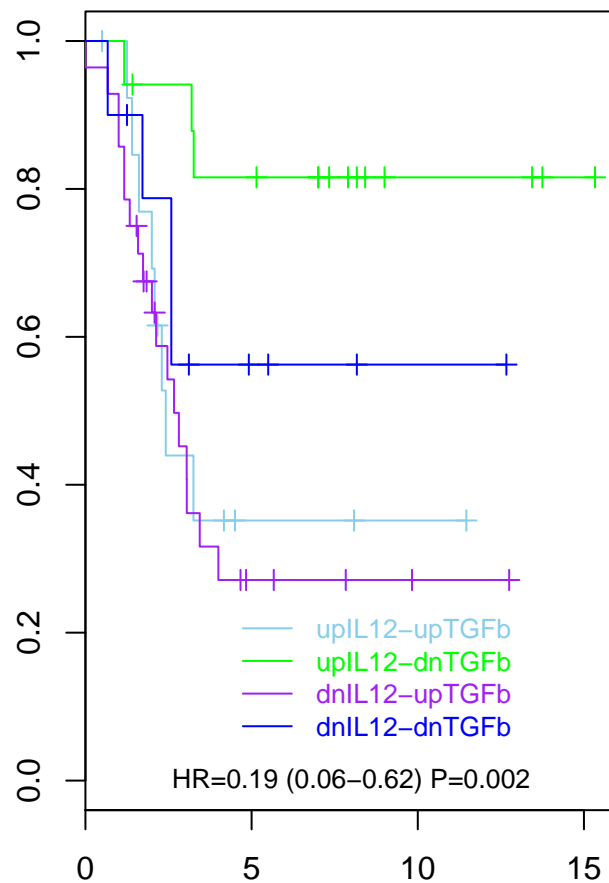

Supplement: Additional file 10 — KM-curves for IL12 and TGFB in ER- subtypes. Kaplan Meier DMFS curves for the four subtypes stratified according to up/down activity of the IL12 and TGFB pathways. Hazard ratio refers to the IL12up-TGFBdn subtype relative to the rest. 95% confidence intervals and log-rank test P-values are given. A) ER- basal samples in Set1. B) ER- HER2+ samples in Set1. [file 1471-2407-10-604-S10.PDF]

**A)**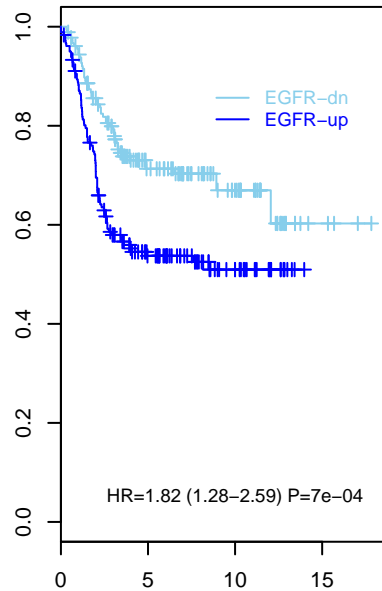**B)**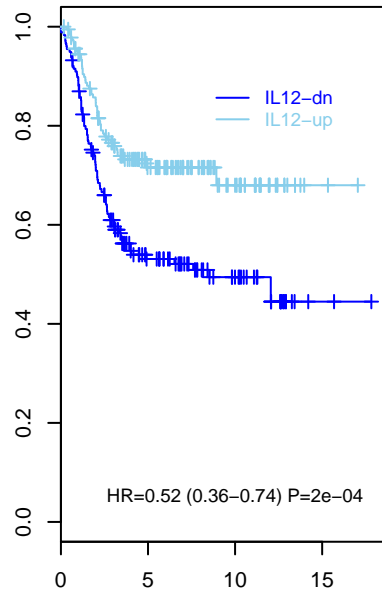**C)**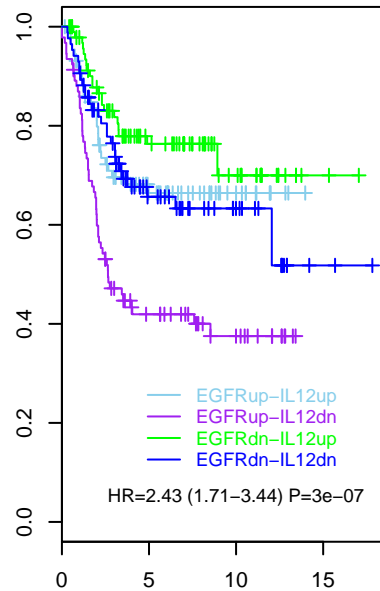**D)**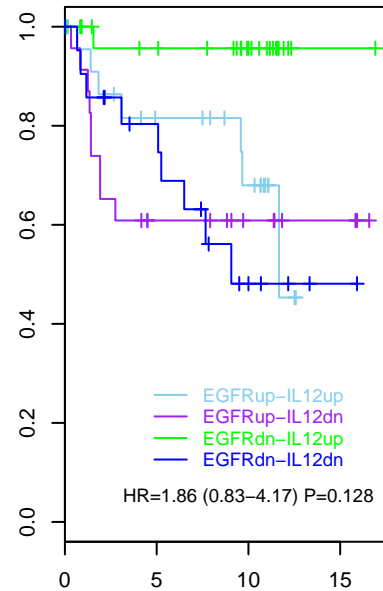

Supplement: Additional file 11 — KM curves for IL12 and EGFR0. A) & B) Kaplan Meier DMFS curves for dichotomised pathway activity levels, for IL12 and EGFR in ER- breast cancer (Set1), respectively. C) Kaplan Meier DMFS curves for the four subtypes stratified according to up/down activity of the IL12 and EGFR pathways in Set1. Hazard ratio refers to the IL12dn-EGFRup subtype relative to the rest. 95% confidence intervals and log-rank test P-values are given. D) As C) but in Set2. [file 1471-2407-10-604-S11.PDF]

**A)****ERpos**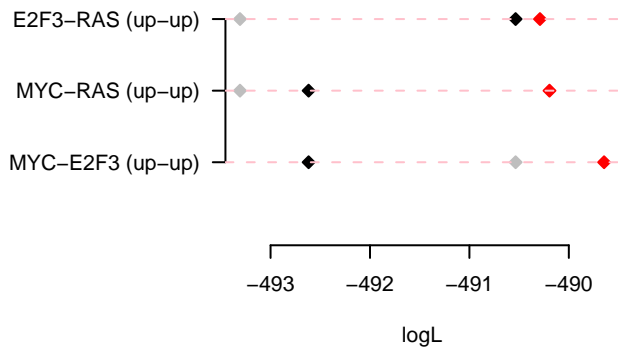**ERneg**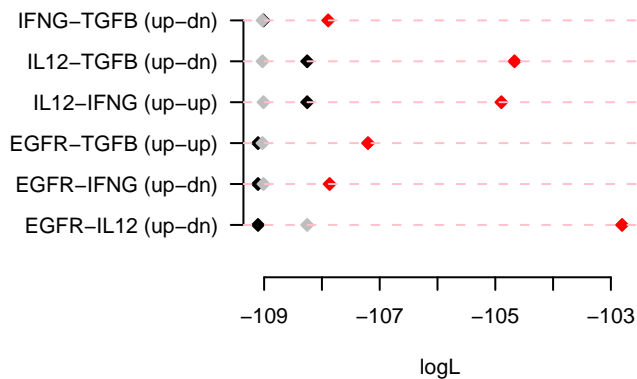**B)**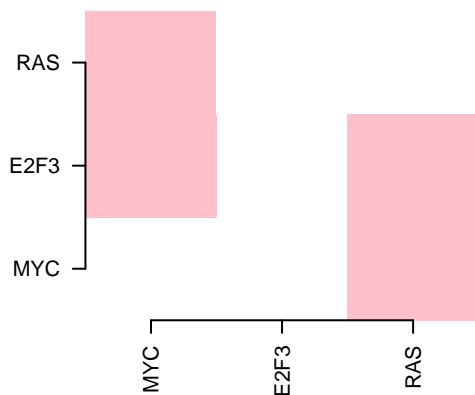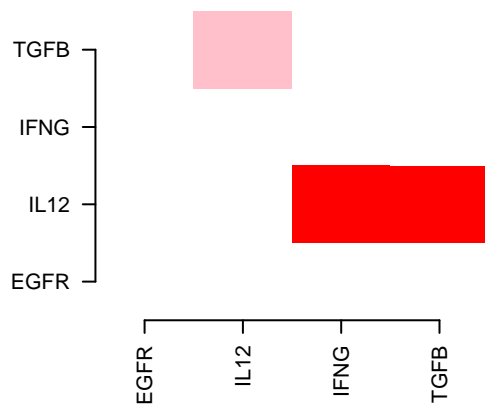**C)**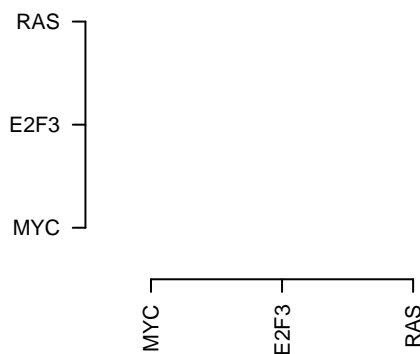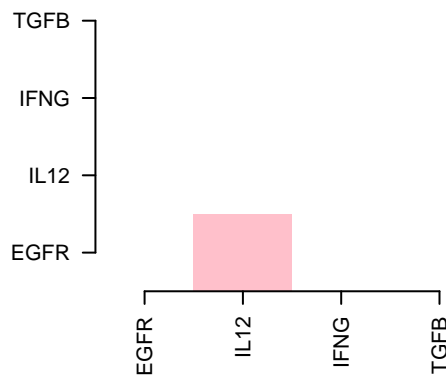

Supplement: Additional file 12 — Synergy outcome maps in Set 2. Validation of module interaction prognostic models in Set2. A) For the pathway modules that correlated with DMFS in the ER positive and negative breast cancer training sets and the corresponding best Boolean interaction regression model, we evaluate the association with prognosis in the validation Set2. x-axis denotes the log-likelihood of the corresponding model. (Black = log-likelihood of model for first pathway in pair, Grey = log-likelihood of model for second pathway in pair, Red = log-likelihood of the best Boolean interaction model as determined from training Set1, pink dashed line highlights those Boolean models with improved log-likelihoods). B) Heatmaps of likelihood ratio test (LRT) p-values comparing nested prognostic models in Set2. Specifically LRT p-value for pathway py on y-axis and pathway px on x-axis is obtained by comparing Cox-regression models with the single pathway px plus non-linear Boolean interaction B(px, py) as predictors against the model with only px as predictor. C) As B), but LRT p-value for pathway py on y-axis and pathway px on x-axis is obtained by comparing Cox-regression models with the single pathway px plus non-linear Boolean interaction B(px, py) as predictors against the model with only B(px, py) as predictor. Color codes: red (P < 0.01), pink (P < 0.05), white (P > 0.05). [file 1471-2407-10-604-S12.PDF]

## ERpos

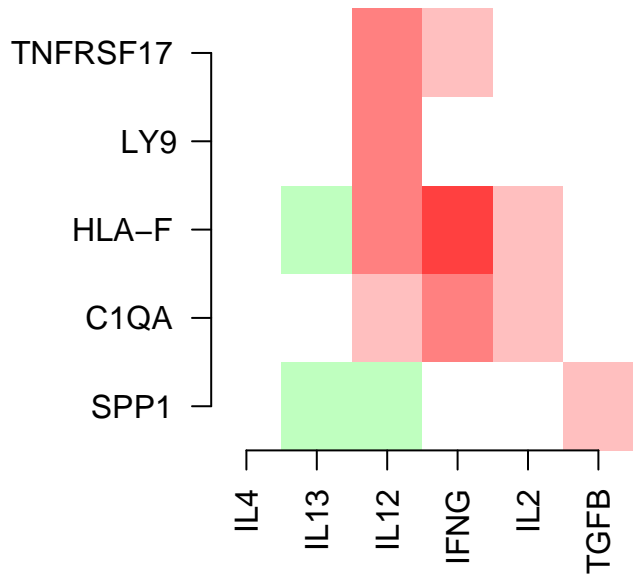

## ERneg

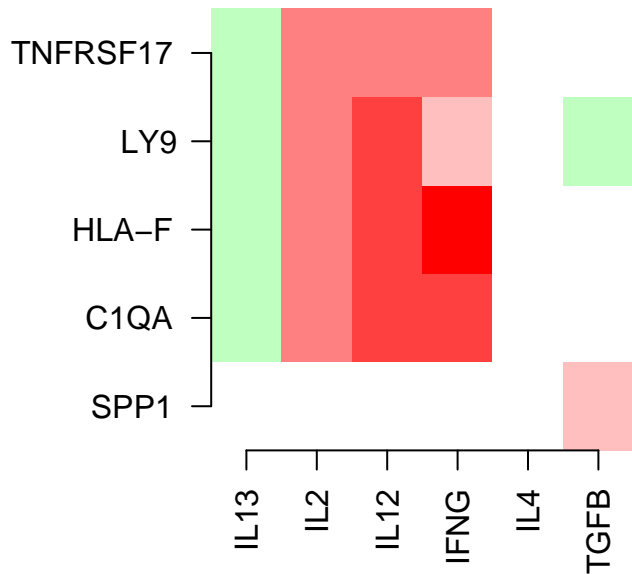

Supplement: Additional file 13 — Relation of pathway modules to IR-module. Correlation heatmaps between activation levels of immune response related pathways and expression levels of the prognostic immune-response (IR) module of [15] in ER positive and ER negative breast cancer (Red = high correlation, White = zero or insignificant correlation, Green = high anti-correlation). Of the seven genes in the IR-module, five were present in Set1. [file 1471-2407-10-604-S13.PDF]
