# Supplementary material for: Improved prognostic classification of breast cancer defined by antagonistic activation patterns of immune response pathway modules
Source: BMC Cancer. 2010 Nov 4;10:604. doi: 10.1186/1471-2407-10-604 (PMC2991308; doi:10.1186/1471-2407-10-604)
Supplement: Additional file 4 — Modularity of pathways. Barplots showing the number (x-axis indexes the module) and sizes (y-axis) of the inferred modules for selected pathways in ER- and ER+ breast cancer, illustrating the modularity structure of pathways. [file 1471-2407-10-604-S4.PDF]

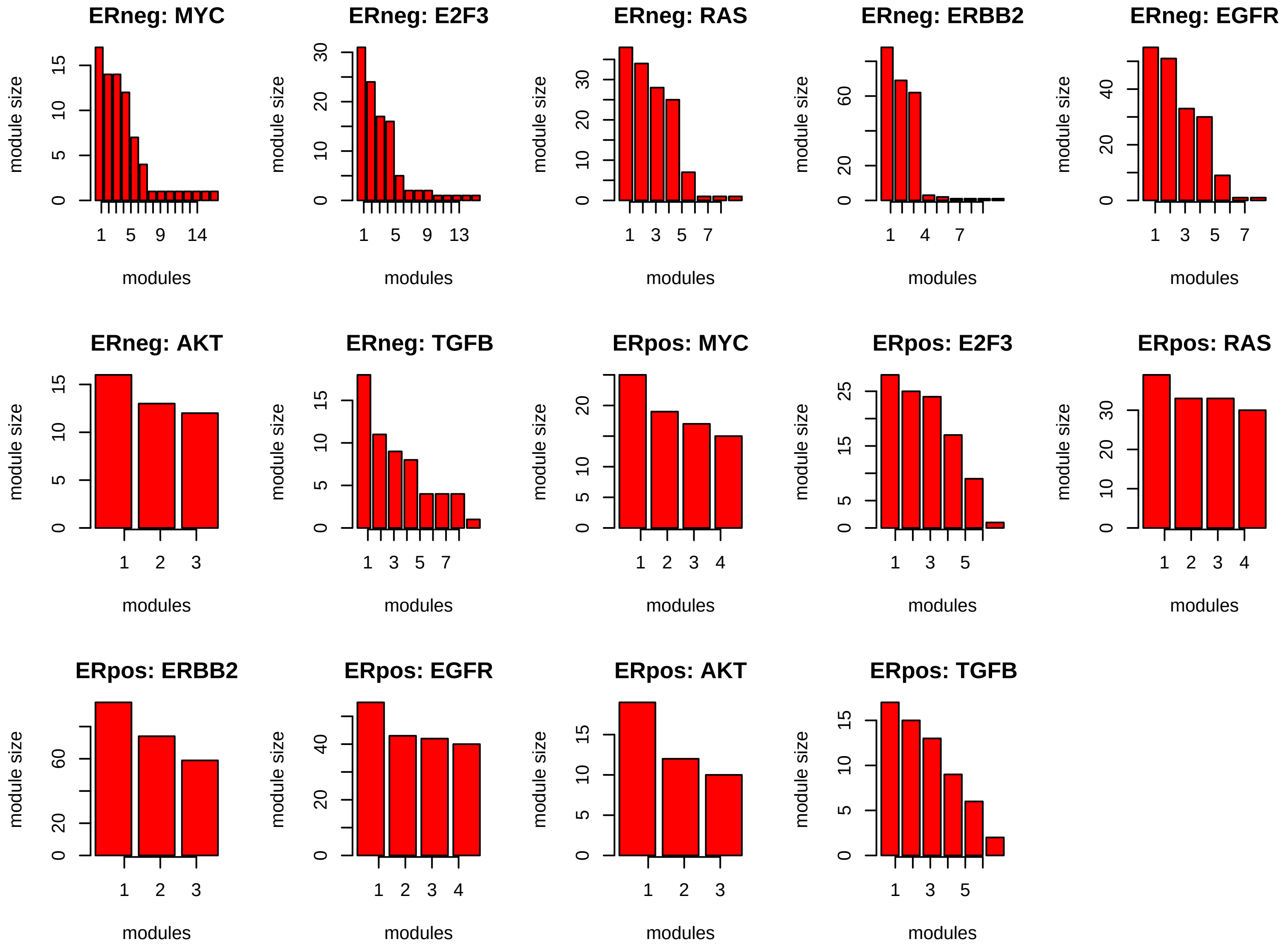
ERneg: MYC
module size
modules
0 5 10 15
1 5 9 14
ERneg: E2F3
module size
modules
0 10 20 30
1 5 9 13
ERneg: RAS
module size
modules
0 10 20 30
1 3 5 7
ERneg: ERBB2
module size
modules
0 20 60
1 4 7
ERneg: EGFR
module size
modules
0 20 40
1 3 5 7
ERneg: AKT
module size
modules
0 5 10 15
1 2 3
ERneg: TGFB
module size
modules
0 5 10 15
1 3 5 7
ERpos: MYC
module size
modules
0 5 10 20
1 2 3 4
ERpos: E2F3
module size
modules
0 5 15 25
1 3 5
ERpos: RAS
module size
modules
0 10 20 30
1 2 3 4
ERpos: ERBB2
module size
modules
0 20 60
1 2 3
ERpos: EGFR
module size
modules
0 20 40
1 2 3 4
ERpos: AKT
module size
modules
0 5 10 15
1 2 3
ERpos: TGFB
module size
modules
0 5 10 15
1 3 5
